# LRP8-dependent cholesterol metabolism modulates mTORC1 signaling and apoptotic pathways in multiple myeloma

**DOI:** 10.1038/s41419-025-07625-w

**Published:** 2025-04-08

**Authors:** Yue Wang, Tianwei Lan, Chi Zhou, Qiongyan Zhang, Peng Liu

**Affiliations:** 1 Department of Hematology, Zhongshan Hospital, Fudan University, Shanghai, China; 2 Department of Pathology, Zhongshan Hospital, Fudan University, Shanghai, China

**Keywords:** Myeloma, Cancer metabolism, Oncogenes, Myeloma

## Abstract

Cholesterol plays a crucial role in tumor metabolism. Studies have shown that the serum cholesterol level of multiple myeloma (MM) patients significantly decreases, probably owing to the augmented uptake by MM cells. Despite its significance for MM, research on its metabolism within MM is limited. Our analysis of clinical data from 703 newly diagnosed MM patients revealed that low serum cholesterol is associated with poor prognosis, and it stems from the elevated cholesterol consumption by MM cells. By exploring the transcriptome and single-cell RNA-seq data of patients with different cholesterol levels in our center, we identified LRP8 as a key regulator of cholesterol metabolism in MM, which is closely related to prognosis and disease stages. We verified the oncogenic role of LRP8 in vitro and in vivo. Knockdown of LRP8 can facilitate apoptosis and cell cycle arrest in MM cells. Meanwhile, we employed mouse xenograft tumor model to replicate the phenomenon that MM cells with high LRP8 expression consume cholesterol, causing low serum cholesterol. Mechanistically, high LRP8 expression enhances cholesterol utilization and uptake by MM cells; LRP8 inhibition reduces cholesterol absorption, further weakening the activity of the cholesterol-dependent mTORC1 pathway in MM cells and inducing apoptosis. Concurrently, it triggers an upregulation of protective autophagy. Further suppression of autophagy can lead to extensive apoptosis of MM cells. Our study reveals that LRP8 regulates cholesterol metabolism in MM cells and influences the processes of cell apoptosis and autophagy through metabolic-related pathways. LRP8 holds potential as a therapeutic target for MM.

## Introduction

During tumorigenesis, tumor cells undergo metabolic reprogramming to meet the energy and material demands. Cholesterol is a vital lipid for cellular function, essential for cell proliferation and differentiation, as well as intracellular signal transduction [1]. Cholesterol primarily circulates in the bloodstream in the form of low-density lipoprotein (LDL) and is internalized by cells through protein-mediated endocytosis [2, 3]. Dysregulation of genes associated with cholesterol metabolism has been identified in various types of tumors, and overexpression of the low-density lipoprotein receptor (LDLR) and related proteins (LRP), responsible for mediating cholesterol uptake, has been implicated in the development and progression of several cancer cell types [4–6].

The survival and proliferation of MM cells have been found to be intricately linked with cholesterol metabolism. Multiple retrospective clinical analyses have demonstrated that serum cholesterol levels are significantly reduced in MM patients, correlating with disease staging, tumor burden, treatment response, and prognosis [7–9]. Furthermore, statins can impede the survival of MM cells by suppressing cholesterol biosynthesis, and enhancing patient prognosis [10, 11]. The observed low serum LDL in MM patients may stem from heightened uptake of LDL by MM cells [8]. Given the elevated energy demands of MM due to its own proliferation and survival requisites as well as the secretion of immunoglobulin and light chains by plasma cells, cholesterol metabolism assumes a pivotal role in supporting these processes. However, despite its significance, research on the implications of cholesterol metabolism for MM cells has been limited. Recently, it has been revealed that cholesterol serves as an essential activator factor for the master growth regulator, mTORC1 kinase [12–14]. Cholesterol facilitates mTORC1 translocation from the cytosol to the lysosomal membrane where it initiates downstream programs for biomass production while inhibiting catabolic metabolism [15]. Moreover, cholesterol plays a crucial role in regulating autophagy in tumor cells [12, 16].

Through an extensive investigation of a large cohort of newly diagnosed MM (NDMM) patients in our center, we have observed a significant reduction in serum cholesterol and LDL levels among MM patients. The decrease in serum cholesterol is associated with the uptake of MM cells and represents a substantial prognostic factor for individuals with MM. Additionally, low-density lipoprotein receptor-related protein 8 (LRP8), a member of the LDLR family, has emerged as a pivotal factor contributing to dysregulated cholesterol metabolism and promoting the progression of MM. LRP8 exerts a significant impact on the metabolism, proliferation and survival of MM cells via cholesterol uptake.

## Results

### The reduction of serum cholesterol levels is related to a poor prognosis for MM patients

**Fig. 1:**
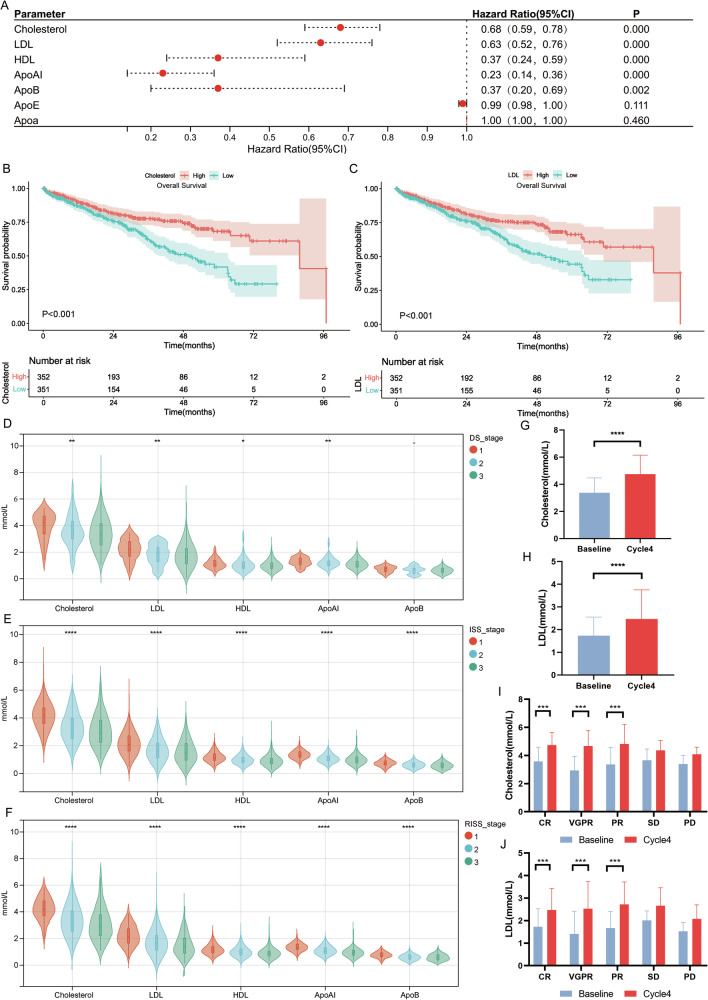
Serum cholesterol levels and the prognosis of patients with multiple myeloma. **A** The results of the univariate COX regression analysis regarding different serum cholesterol indicators and the OS of 703 newly diagnosed MM patients (NDMM patient data for cholesterol and prognosis studies were from 2014 to 2021. All received first-line therapy with core drugs of proteasome inhibitors and/or immune modulators. Only ineligible-for-transplantation patients were included. 703 patients were included. **B**, **C** The median values of patients’ serum cholesterol (**B**) and LDL (**C**) were utilized as the cut-off thresholds to stratify 703 patients into two groups, and the Kaplan–Meier survival curves of OS for patients in disparate groups were presented. **D**–**F** The disparities in serum cholesterol indicators among patients with different DS(**D**), ISS(**E**) and R-ISS(**F**) stages. **G**, **H** The serum cholesterol(**G**)/LDL(**H**) levels of 355 newly diagnosed MM patients at baseline and after 4 cycles of treatment. (A total of 355 patients were recruited from NDMM patients who received continuous first-line treatment for 4 cycles from 2016 to 2021 and had complete efficacy evaluations after the 4 cycles.) The results indicated that the patient’s serum cholesterol and LDL levels were significantly elevated after 4 cycles of treatment. **I**, **J** The serum cholesterol(**I**)/LDL(**J**) levels of 355 newly diagnosed MM patients with different treatment responses at the baseline and after 4 cycles of treatment. Among patients with treatment response rates of PR, VGPR, and CR, serum total cholesterol and LDL were significantly elevated, whereas no significant elevation of serum total cholesterol and LDL was observed in patients with treatment response rates of SD and PD).

We analyzed the prognostic value of serum lipids in 703 NDMM patients in our center (Baseline characteristics in Supplementary Table 1). We analyze the prognostic value of seven parameters, including serum total cholesterol, LDL, high-density lipoprotein (HDL), apolipoprotein A-I (ApoAI), apolipoprotein B (ApoB), apolipoprotein E (ApoE) and apolipoprotein a (Apoa). The results showed that cholesterol, LDL, HDL, ApoAI, and ApoB were prognostically significant (Fig. 1A).

Then, we stratified the patients into two groups based on the median values of cholesterol and LDL. The survival analysis revealed that patients with high serum cholesterol (*P* < 0.001) and LDL (*P* < 0.001) exhibited a longer overall survival (OS) (Fig. 1B, C).

We posited two potential causes for the decreased serum cholesterol in MM patients: the insufficient nutrient intake of patients and the cholesterol consumption resulting from tumor growth. However, correlation analysis between low serum cholesterol and low BMI ( < 18.5) showed no significant association (*p* = 0.895), ruling out malnutrition as a primary cause.

As the DS staging primarily evaluates the tumor burden of myeloma patients, we analyzed the cholesterol levels of patients at different DS stages. The findings demonstrated a close association between cholesterol levels and DS stage (Fig. 1D). We also calculated the relationship between cholesterol level and ISS stage (Fig. 1E) and R-ISS stage (Fig. 1F), finding that serum cholesterol level decreased as the stage increased.

The relationship between treatment response and serum cholesterol was also examined. Through the analysis of patients after four treatment cycles, it was revealed that serum cholesterol significantly increased in patients with therapeutic efficacies of PR (*p* < 0.001), VGPR (*p* < 0.001), and CR (*p* < 0.001), while no significant changes were observed in patients with SD (*p* = 0.066) or PD (*p* = 0.127). Similar trends were observed for LDL levels (Fig. 1G–J).

### LRP8 is a crucial regulatory gene for cholesterol metabolism in MM cells and influences the prognosis of patients

**Fig. 2:**
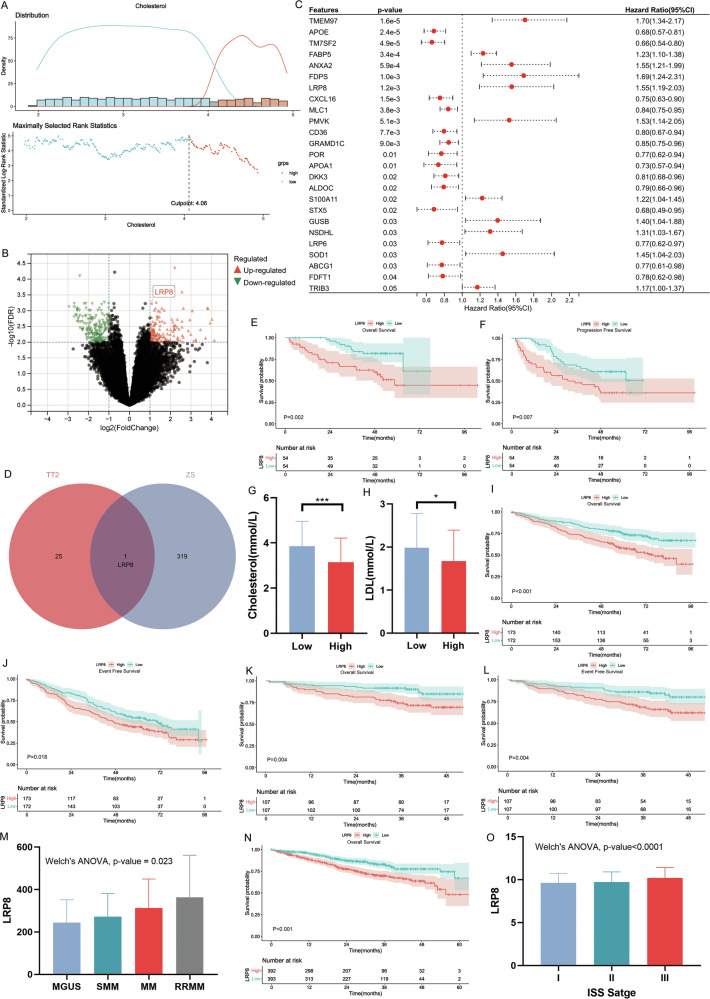
LRP8 serves as the critical gene modulating cholesterol metabolism in MM cells and is associated with the prognosis, disease stage, and staging of patients. **A** The determination of the cutoff value of serum cholesterol level (Determined via the “cutpoint” function of the “Survival” package). **B** The volcano plot of differentially expressed genes in the plasma cells from 20 MM patients with conspicuously reduced serum cholesterol and 20 MM patients with normal serum cholesterol (|logFC | > 1, FDR < 0.05). **C** Univariate Cox analysis identified genes related to cellular cholesterol uptake and metabolism that had prognostic value in the TT2 cohort. **D** A total of 25 genes related to cholesterol metabolism and with prognostic significance were identified in the TT2 cohort. A total of 319 differentially expressed genes in the plasma cells of 40 patients with diverse serum cholesterol levels were determined. The intersection gene was LRP8. **E**, **F** The KM survival curves of OS(**E**) and PFS(**F**) for patients in our center with different expression levels of LRP8. **G**, **H** The serum cholesterol(**G**) and LDL(**H**) levels of patients presenting with different expression level of LRP8. **I**, **J** The KM survival curves of OS(**I**) and EFS(**J**) for patients in TT2 cohort with different expression levels of LRP8. **K**, **L** The KM survival curves of OS(**K**) and EFS(**L**) for patients in TT3 cohort with different expression levels of LRP8. **M** The expression level of LRP8 in plasma cell neoplasms at diverse stages in the GSE6477 cohort. LRP8 shows a gradually ascending trend in line with the disease stage progression. **N** The KM survival curves of OS for patients in MMRF cohort with different expression levels of LRP8. **O** The expression level of LRP8 in plasma cell neoplasms at different ISS stages in the MMRF cohort. LRP8 shows a steadily rising trend as tumor stages progress).

Using serum cholesterol levels from 703 patients, we established an optimal cholesterol reduction cutoff of < 4.06 mmol/L (Fig. 2A). To explore key regulatory genes in hypercholesterolemic metabolism, bone marrow samples from 20 newly diagnosed multiple myeloma (NDMM) patients with low serum cholesterol (< 3.06 mmol/L, > 1 mmol/L below the cutoff) and 20 with high serum cholesterol (> 5.06 mmol/L, > 1 mmol/L above the cutoff) were analyzed. RNA sequencing after CD138+ magnetic bead sorting identified 319 differentially expressed genes (via DESeq2) (|logFC | >1, FDR < 0.05) (Fig. 2B).

From MSigDB [17], 138 cholesterol metabolism-related genes were screened for prognostic value in the TT2 (GSE24080) cohort, yielding 25 significant genes (Fig. 2C). Subsequently, the intersection of the two screening methods was taken, and it was found that LRP8 was a gene involved in cholesterol metabolism and had significant prognostic value (Fig. 2D). Then, further expanded validation was carried out in the specimens of a cohort of 108 NDMM patients in our center (Baseline characteristics shown in Supplementary Table 2). Taking the median value of LRP8 expression, the patients were classified into two groups. It was discovered that patients in high-LRP8-expression group had significantly shorter OS (*p* = 0.002) (Fig. 2E) and progression-free survival (PFS) (*p* = 0.007) (Fig. 2F). Meanwhile, the serum cholesterol/LDL level of patients in the high-LRP8-expression group was lower (Fig. 2G, H). Then, separate validations were conducted in the TT2 and TT3 cohorts. The findings indicated that a high expression level of LRP8 was associated with shorter event-free survival (EFS) and OS (Fig. 2I–L). Simultaneously, the expression level of LRP8 at distinct stages of plasma cell neoplasms was verified (GSE6477). The results disclosed that during the evolutionary process of monoclonal gammopathy of undetermined significance (MGUS), smoldering multiple myeloma (SMM), NDMM, and relapsed/refractory multiple myeloma (RRMM), LRP8 exhibited a continuously ascending tendency (Fig. 2M). Concurrently, the prognostic value of LRP8 was validated in the MMRF database (Fig. 2N), and it was discovered that the expression level of LRP8 gradually escalated with the increase in the ISS stage (Fig. 2O).

### Single-cell transcriptome confirmed that serum cholesterol reduction originated from high cholesterol metabolism of MM, with LRP8 identified as a key differentially expressed gene

**Fig. 3:**
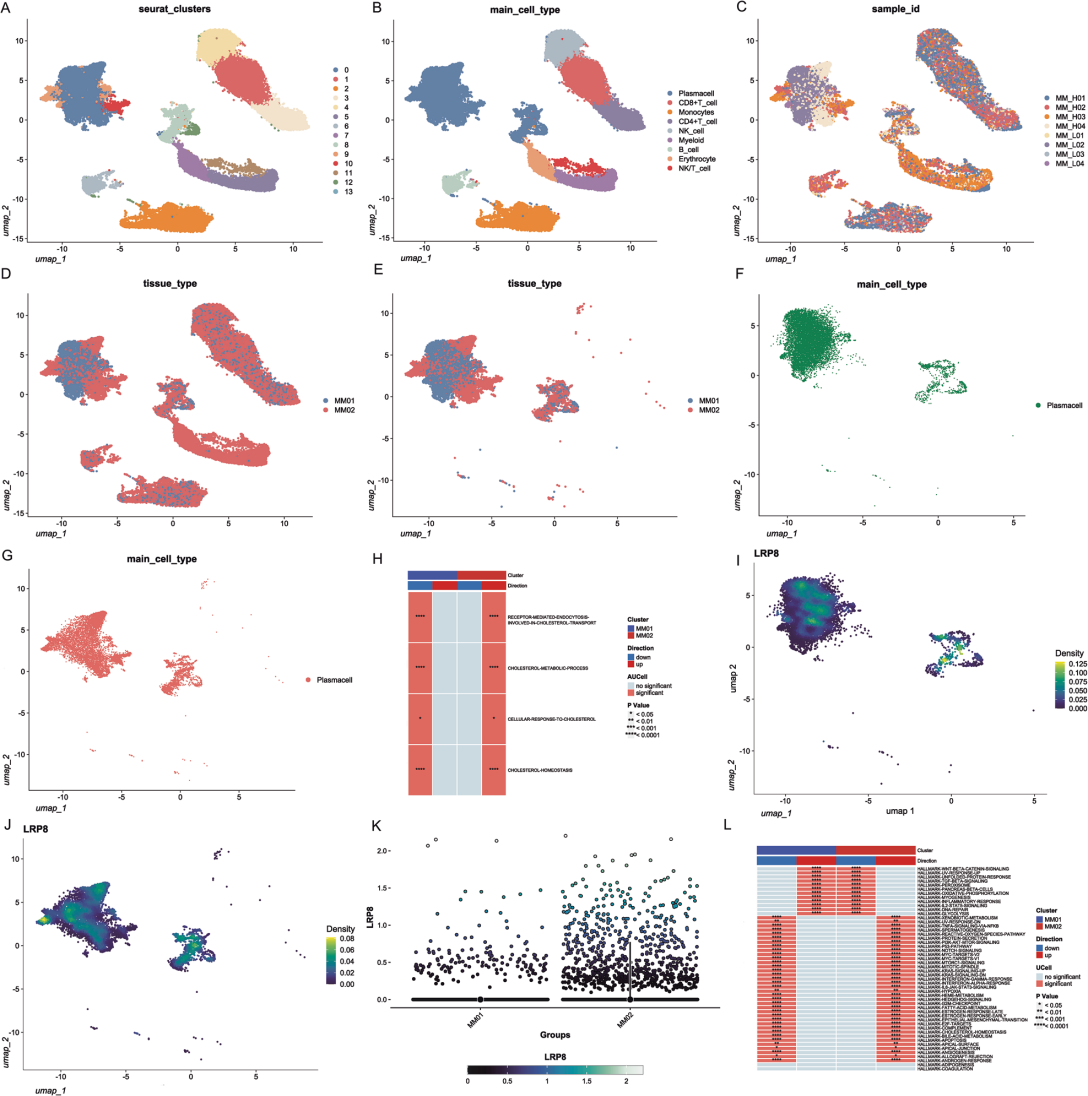
The findings of bone marrow single-cell RNA-seq in newly diagnosed multiple myeloma patients presenting with distinct serum cholesterol levels. The samples encompassed bone marrow specimens from 4 patients with conspicuously decreased serum cholesterol (less than 3.06 mmol/L) and 4 patients with normal serum cholesterol (greater than 5.06 mmol/L). Herein, MM01 was the subgroup featuring normal serum cholesterol, while the MM02 cohort belonged to the subgroup with significantly lowered serum cholesterol. MM_H01-MM_H04 were assigned to the MM01 subgroup (normal cholesterol), and MM_L01-MM_L04 to the MM02 subgroup (low cholesterol). **A** Cell clustering results from single-cell RNA-seq in eight newly diagnosed MM patients. **B** The cell subpopulation profiles of bone marrow samples from eight patients with NDMM. **C** The distribution of bone marrow samples from eight NDMM patients. **D** The distribution of bone marrow samples from different group. **E** The plasma cells in the bone marrow specimens of patients from different groups. **F**, **G** The plasma cells in the bone marrow specimens of patients from MM01 subgroup(**F**) and MM02 subgroup(**G**). **H** The activation of cholesterol-related pathways in the plasma cells of patients in different subgroups. The cholesterol metabolism in the plasma cells of patients in the low serum cholesterol group (MM02) was significantly enhanced, suggesting that the low serum cholesterol resulted from the high consumption by MM cells. **I**, **J** The expression level of LRP8 in the plasma cells of patients within the MM01(**I**) and MM02(**J**) subgroup. **K** The expression level of LRP8 in the plasma cells of patients in the MM02 subgroup was significantly elevated. **L** The activation states of the related pathways of the hallmark gene set in the plasma cells of patients in different subgroups. A significant upregulation of the mTORC1 pathway and the cholesterol uptake pathway was observed in MM02).

We conducted single-cell transcriptome sequencing (scRNA-seq) on 4 NDMM patients with high serum cholesterol (MM01) and 4 NDMM patients with low serum cholesterol (MM02) (with the screening criteria remaining consistent as before). We implemented dimensionality reduction, clustering and cell identification on the cells (Fig. 3A–D) and screened out the plasma cell subpopulations (Fig. 3E–G). The activities of cholesterol metabolism pathways in patients of different groups were analyzed using irGSEA. The results revealed that the levels of cholesterol endocytosis, cholesterol metabolism, cholesterol homeostasis and cellular responses to cholesterol in the low serum cholesterol group (MM02) were significantly elevated (Fig. 3H). This further substantiated that the consumption attributed to the high cholesterol metabolism of MM cells was the reason for low serum cholesterol. Concurrently, we also ascertained that the expression level of LRP8 in MM cells in the MM02 group was significantly higher than that in the MM01 group (*P* < 0.001) (Fig. 3I–K).

### The expression level of LRP8 influences the cholesterol uptake capacity of MM cells

**Fig. 4:**
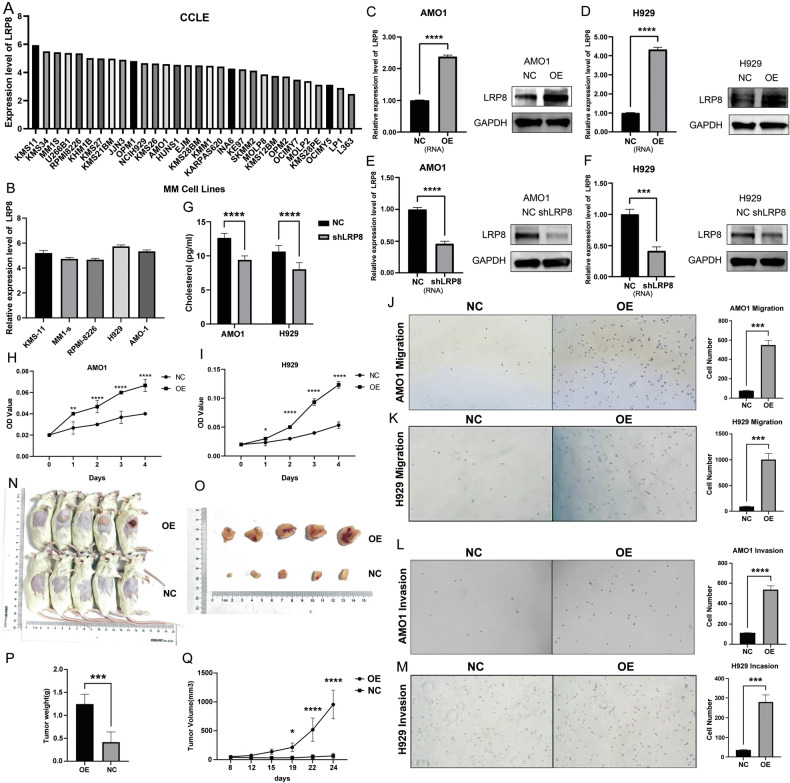
LRP8 significantly enhances the proliferation, invasion and migration of myeloma cells. **A** The expression level of LRP8 in different MM cell lines in the CCLE database. **B** The expression level of LRP8 in various MM cell lines was assessed by RT-PCR. **C**, **D** RT-PCR and WB were adopted to assess the expression level of LRP8 in the normal control (NC) and LRP8-overexpression (OE) AMO1(**C**)/H929(**D**) cell lines. **E**, **F** RT-PCR and WB were adopted to assess the expression level of LRP8 in the NC and LRP8-knockdown (shLRP8) AMO1(**E**)/H929(**F**) cell lines. **G** ELISA was utilized to determine the cholesterol content in the NC and LRP8-knockdown (shLRP8) AMO1/H929 cell lines. The downregulation of the expression level of LRP8 conspicuously diminished the cholesterol uptake capability of MM cells. **H**, **I** The proliferation capacity of NC and LRP8-OE AMO1(**H**)/ H929(**I**) cell lines (2000 cells/well) were assessed via the CCK8 assay. **J**, **K**. Transwell assays were conducted to assess the migratory potential of NC and LRP8-OE AMO1 (**J**) and H929 (**K**) cell lines, demonstrating a significant enhancement in migration ability in the LRP8-OE group. **L**, **M** The invasion ability of NC and LRP8-OE AMO1(**L**)/H929(**M**) cell lines were assessed via the Transwell assay. The invasion ability of MM cells in LRP8-OE group was significantly strengthened**. N**–**Q** T Subcutaneous xenograft mouse model: Tumor growth (**N**, **O**), weight (**P**), and volume (**Q**) were significantly increased in LRP8-OE groups compared to controls).

In the CCLE database, LRP8 manifested a relatively high expression level in the majority of MM cell lines (Fig. 4A). Subsequently, qPCR confirmed LRP8 expression in KMS-11, MM1-S, RPMI-8226, H929, and AMO1 cell lines, with the highest levels observed in AMO1 and H929 (Fig. 4B). These two lines were selected for subsequent studies. LRP8-overexpressing (LRP8-OE) lentivirus and normal control (NC) lentivirus, as well as LRP8-konckdown (shLRP8) lentivirus and NC lentivirus were employed to infect the AMO1 and H929 cell lines. We adopted qRT-PCR and WB to detect the expression level of LRP8 in different cell lines (Fig. 4C–F). Cholesterol levels in cell lines were measured by ELISA (Fig. 4G). The results indicated that LRP8 expression levels were positively correlated with cholesterol uptake capacity in MM cells.

CCK-8 assays revealed that LRP8 overexpression significantly enhanced MM cell proliferation (Fig. 4H, I). Transwell assays demonstrated increased migration and invasion capacities in LRP8-OE cells (Fig. 4J–M). Then, we utilized the LRP8-OE and NC AMO1 cell lines to construct the subcutaneous xenograft tumor model in BNGD mice through subcutaneous injection. Subsequent analyses indicated that the tumor growth in the LRP8-OE group mice was faster (Fig. 4N–Q).

**Fig. 5:**
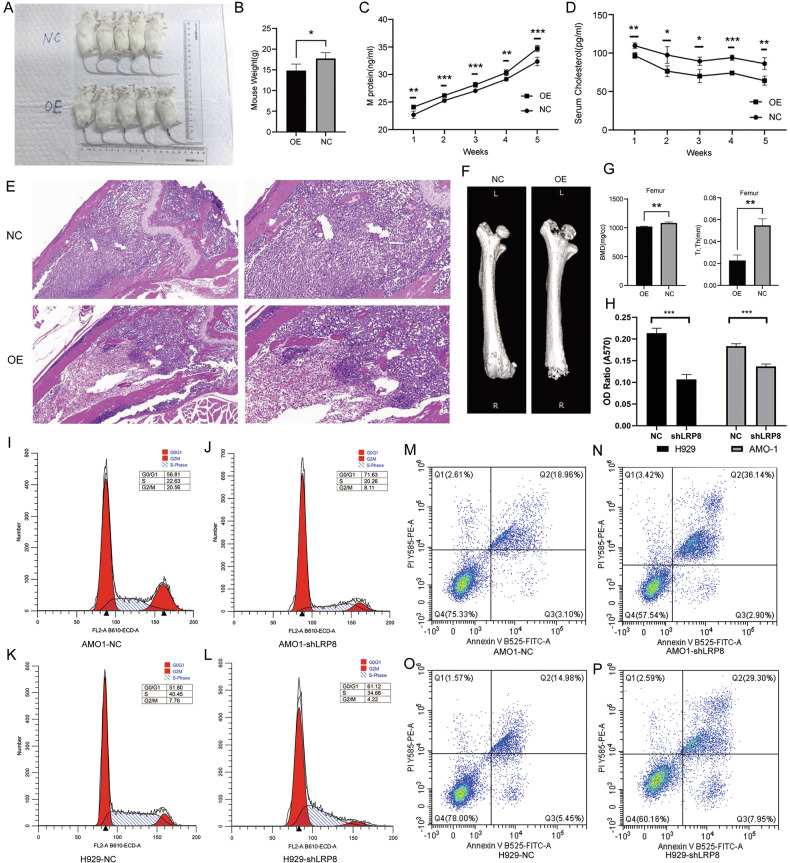
LRP8 promotes the occurrence of hypocholesterolemia in mouse xenograft tumor, and downregulation of LRP8 induces cell cycle arrest and apoptosis in MM cells. **A**, **B** Tail vein-injected mouse xenograft tumor model established with the LRP8-OE and NC AMO1 cell lines revealed that the body weight of mice in the LRP8-OE group decreased significantly. **C** M protein levels in the mouse xenograft model were measured by ELISA. Results showed that serum M protein levels increased more rapidly in the LRP8-OE group, indicating faster tumor growth and a higher tumor burden. **D** ELISA assays performed on mouse xenograft tumor models disclosed that with the proliferation of MM cells, mice presented a continuous lowering of serum cholesterol levels, and the serum cholesterol levels in the LRP8-OE group mice were even lower. **E** The HE staining images of femoral sections in the mouse xenograft tumor model disclosed that MM cells successfully homed and colonized in the bone marrow, and more prominent tumor colonization and a greater tumor burden were noted in the LRP8-OE group. **F**, **G** The micro-CT assessment of the femoral bone in the mouse xenograft tumor model demonstrated that the bone mineral density and trabecular thickness of the femur in the LRP8-OE group were significantly diminished. **H** The MTT assay (24 h) indicated that the apoptosis of MM cell lines was significantly enhanced subsequent to knockdown of LRP8. **I**–**L** Flow cytometry demonstrated that knockdown of LRP8 in AMO1(**I**, **J**) and H929 (**K**, **L**) cell lines gave rise to cell cycle arrest, accompanied by a marked reduction in the proportion of cells in the G2/M phase. **M**–**P** Flow cytometry indicated that knockdown of LRP8 in AMO1 (**M**, **N**) and H929 (**O**, **P**) cell lines led to a substantial increase in apoptotic cells).

In an attempt to investigate the impact of LRP8 expression levels on the biological behavior of myeloma cells, we utilized the LRP8-OE and NC AMO1 cell lines to construct a BNGD mouse xenograft tumor model via tail vein injection. We employed ELISA detection to quantify serum M protein level and serum cholesterol level of mice. We found that, compared with the mice in the NC group, mice in the LRP8-OE group were lighter (*p* = 0.014) (Fig. 5A, B). The serum M protein level of mice showed an overall upward trend over time, and the increase was significantly more prominent in LRP8-OE group than in NC group (Fig. 5C). Concurrently, the results of ELISA assay revealed that serum cholesterol of mice exhibited an overall downward trend over time, and the decline was significantly faster in LRP8-OE group than in NC group (Fig. 5D). Histological analyses (HE staining) of femurs confirmed MM cell infiltration, with faster growth in LRP8-OE mice (Fig. 5E). Micro-CT imaging demonstrated exacerbated bone destruction in LRP8-OE mice, with significantly lower bone mineral density (BMD, *p* = 0.005) and trabecular thickness (Tr.Th, *p* = 0.002) compared to controls (Fig. 5G).

### Downregulation of LRP8 induces growth suppression, cell cycle arrest, and apoptosis in MM cells

Since LRP8 is generally highly expressed in myeloma, we thus aim to further observe the influence that interference with LRP8 exerts on myeloma. MTT assays found that the cell viability of the shLRP8 cell lines decreased significantly (Fig. 5H). Then, the cell cycle of shLRP8 cells was examined by flow cytometry. The results revealed that knockdown of LRP8 led to abnormal cell cycle arrest. Notably, the G2/M phase cells of AMO1 and H929 cells decreased significantly (Fig. 5I–L). We further detected the cell apoptosis by cytometry flow, and the results indicated that suppression of LRP8 induced stronger cell apoptosis (Fig. 5M–P).

### Suppression of LRP8 inhibits the mTORC1 signaling pathway and increases the level of autophagy in MM cells

**Fig. 6:**
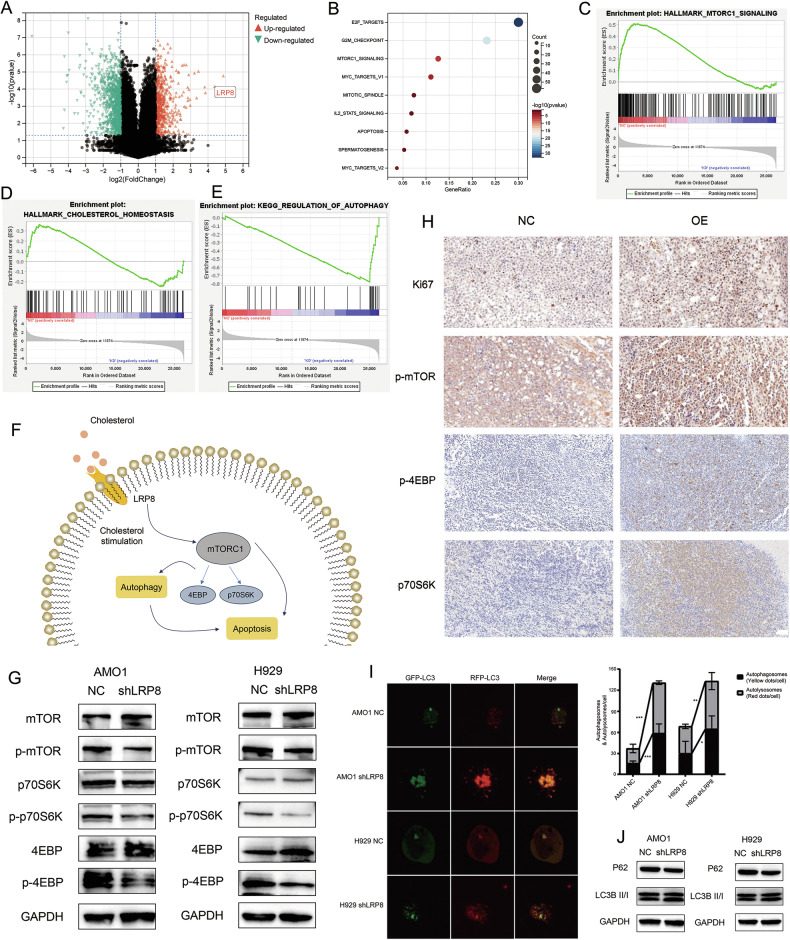
The mTORC1 pathway and regulation of autophagy pathways were significantly activated in shLRP8 MM cell lines. **A** The volcano plot of differentially expressed genes derived from RNA-seq detection comparing the NC AMO1 cell line with the shLRP8 AMO1 cell line (|logFC | > 1, *p* < 0.05). **B** KEGG pathway enrichment analysis of differentially expressed genes in the NC AMO1 cell line compared with the shLRP8 AMO1 cell line. **C–E** The GSEA analysis results of mTORC1 (**C**), cholesterol homeostasis (**D**) and regulation of autophagy (**E**) and pathways in the NC and shLRP8 AMO1 cell lines. Notably, the mTORC1 pathways, regulation of autophagy pathways and cholesterol homeostasis pathways in the AMO1 cell lines of shLRP8 underwent significant alterations. In the cell lines of the NC group, both the mTORC1 pathway and the cholesterol homeostasis pathway were conspicuously upregulated in comparison with those of the shLRP8 group, whereas the regulation of autophagy pathway was significantly downregulated. **F** Hypothetical mechanism diagram of LRP8 influencing intracellular signaling pathways in MM cells. LRP8 affects mTORC1 pathway via cholesterol uptake, and subsequently impacts the autophagy level and apoptosis of cells. **G** In the WB assay of the AMO1 and H929 cell lines, it was verified that the MTORC1 pathway was conspicuously inhibited in the shLRP8 cell lines. In contrast to the NC cell lines, the protein quantities of 4EBP, mTOR, and p70S6K were analogous in the shLRP8 cell lines, while the protein expression levels of p-4EBP, p-mTOR, and p-p70S6K were significantly decreased. **H** Immunohistochemical examination of mouse xenograft tumors indicated that the MTORC1 pathway in the tumors of the LRP8-OE group of mice was significantly activated. **I** Immunofluorescence analysis of autophagic flux indicated that the autophagy level was significantly elevated in shLRP8 AMO1 and H929 cell lines compared with that in the NC AMO1 and H929 cell lines. **J** WB assays revealed that the protein level of P62 in the shLRP8 AMO1 and H929 cell lines declined significantly, while the protein level of LC3BII/I increased significantly, indicating that the level of autophagy was upregulated).

We conducted RNA-seq on the NC and shLRP8 cell lines (Fig. 6A). The KEGG pathway analysis (Fig. 6B) indicated that knockdown of LRP8 could notably down-regulate the mTORC1 signaling pathway, the E2F pathway, and the G2M checkpoint pathway, etc. In addition, there are significant differences between the NC and shLRP8 cell lines in the mTORC1 pathway, cholesterol metabolism pathways and regulation of autophagy pathway (Fig. 6C–E). Based on the modifications of the metabolism-based signaling pathways, we propose that LRP8 impacts the activation of the cholesterol-related mTORC1 signaling pathway through regulating cholesterol uptake, and affects autophagy, thereby altering the proliferation and apoptosis features of cells (Fig. 6F).

Then, we detected the expression levels of marker proteins in the mTORC1 signaling pathway, including mTOR, 4EBP, and p70S6K, as well as phosphorylated mTOR (p-mTOR), phosphorylated 4EBP (p-4EBP), and phosphorylated p70S6K (p-p70S6K). The results showed that the expression levels of p-mTOR, p-4EBP, and p-p70S6K in shLRP8 cell lines were significantly decreased (Fig. 6G). Meanwhile, we conducted IHC on mouse xenograft tumors and found that the expression level of the mTORC1 signaling pathway significantly increased in LRP8-OE group (Fig. 6H). Furthermore, in scRNA-seq data, a significant upregulation of the mTORC1 pathway and the cholesterol uptake pathway was also observed in the high LRP8-expression group (MM02) (Fig. 3L).

Then, we infected cells with RFP-GFP-LC3 lentivirus and employed confocal fluorescence microscopy to detect the autophagic flux. The results indicated that the numbers of autophagosomes and autolysosomes significantly increased in shLRP8 AMO1 (*p* < 0.001) and shLRP8 H929 (*p* = 0.024) (Fig. 6I) cells. Concurrently, we discerned that in the shLRP8 cell lines, the expression of LC3BII/I was significantly enhanced, while P62 was significantly decreased, inferring that the autophagy level was significantly elevated (Fig. 6J).

### Suppression of LRP8 inhibits the mTORC1 signaling pathway via cholesterol depletion, thereby resulting in an increase in autophagy

**Fig. 7:**
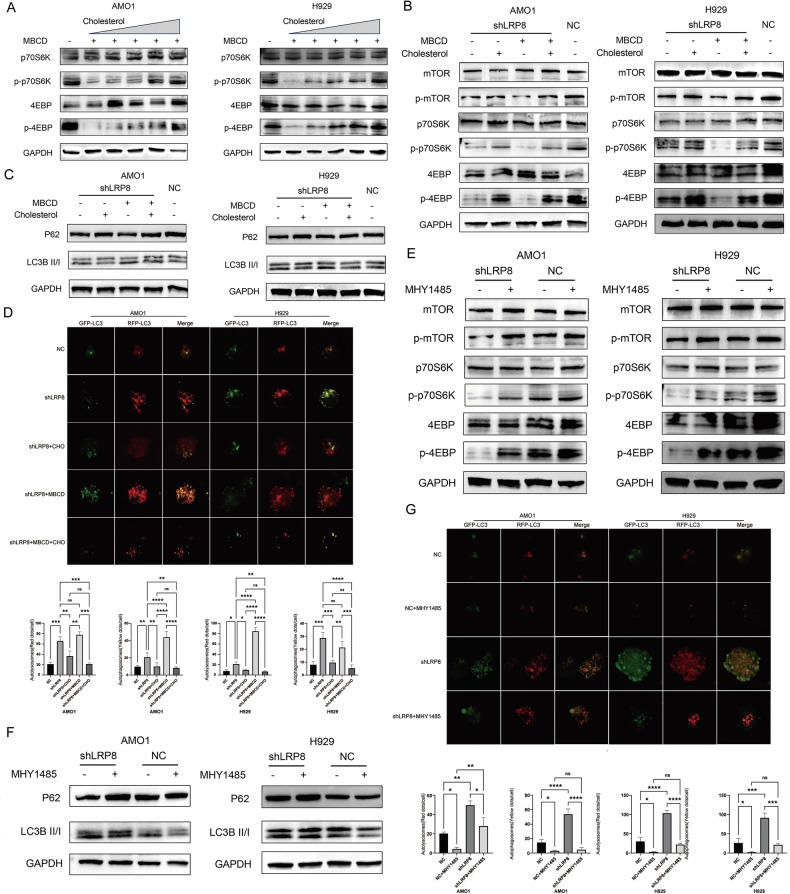
In MM cells, depletion of LRP8 affects cholesterol uptake, subsequently down-regulating the mTORC1 pathway and up-regulating the autophagy level. **A** NC AMO1 and H929 cells were treated with 50 μM MBCD for 2 h to deplete cholesterol in cells, followed by culturing the cells with different concentrations of cholesterol for 24 h. The dosages of cholesterol supplementation are 0 μM, 40 μM, 60 μM, 80 μM, and 100 μM, respectively, in the sequence from left to right. WB analysis revealed that the activation of the mTORC1 pathway in the AMO1 and H929 cell lines was significantly associated with the intake of cholesterol. **B** shLRP8 AMO1 and H929 cells were processed as follows: untreated group, cholesterol group (treated with 100 μM cholesterol for 24 h), MBCD group (treated with 50 μM cholesterol for 2 h), and MBCD + cholesterol group (treated with 50 μM cholesterol for 2 h followed by 100 μM cholesterol for 24 h). NC cells were used as the control. WB detection results showed that compared with the NC group, the expression level of mTORC1 pathway in the “untreated group” of shLRP8 cell lines was significantly decreased. In the shLRP8 cell lines, the expression level of mTORC1 signaling in the “cholesterol group” was significantly higher than that in the “untreated group”. The expression level of mTORC1 signaling pathway in the “MBCD group” was the lowest. Additionally, the addition of cholesterol reversed the down-regulation of the mTORC1 signaling pathway in the MBCD group. **C** The expression ratio of LC3B II/I in the NC group cells was significantly lower than that in the “untreated group” shLRP8 cells. In shLRP8 cell lines, compared with “untreated group”, expression of LC3B II/I in “cholesterol group” was significantly decreased, with an increase in the protein expression of P62; the expression of LC3B II/I in the “MBCD group” was significantly higher than that in the “untreated group”, indicating that cholesterol depletion further enhanced cell autophagy; the expression of LC3B II/I in the “MBCD + cholesterol group” was significantly lower than that in the “MBCD group”, and the protein expression of P62 was increased, suggesting that the addition of cholesterol could reverse the promotion effect of MBCD on autophagy. **D** Autophagic flux was assessed via immunofluorescence. The outcomes manifested that, in contrast to the NC group, the counts of autophagosomes and autolysosomes in shLRP8 cells within distinct cholesterol treatment groups augmented. Upon addition of cholesterol to shLRP8 cells, the quantities of autophagosomes and autolysosomes declined. When cholesterol was depleted by MBCD, the numbers of autophagosomes and autolysosomes escalated. The “MBCD + cholesterol” treatment reversed the autophagy upregulation witnessed in the cells treated with MBCD. **E** In shLRP8 cell lines, after stimulation with MHY1485, the expression levels of p-mTOR, p-p70s6k and p-4ebp were significantly enhanced. **F** Upon treatment of cells with MHY1485, the expression ratio of LC3B-II/I was decreased, and the expression level of P62 protein was increased. This result implies that the autophagy process of the cells is obstructed. **G** Autophagic flux was determined by immunofluorescence, showing that the numbers of autophagosomes and autolysosomes within cells were conspicuously decreased following MHY1485 stimulation).

Firstly, NC AMO1 and H929 cells were treated with methyl-β-cyclodextrin (MBCD) for 2 h to deplete cholesterol, followed by 24 h culture with varying cholesterol concentrations. Cholesterol depletion significantly downregulated mTORC1 pathway proteins, while cholesterol supplementation restored p-p70S6K and p-4EBP1 levels in a dose-dependent manner (Fig. 7A).

Then, the shLRP8 AMO1 and H929 cells were processed as follows and divided into four groups: untreated group, cholesterol group (100 μM cholesterol for 24 h), MBCD group (50 μM MBCD for 2 h), MBCD + cholesterol group (50 μM MBCD for 2 h followed by addition of 100 μM cholesterol for 24 h); untreated NC cells were utilized as controls. The results indicated that compared with the NC group, the expression level of mTORC1-related proteins in the “untreated group” of shLRP8 cell lines was significantly decreased. In shLRP8 cell lines, the expression level of mTORC1-related proteins in “cholesterol group” was significantly increased compared to “untreated group”. Concurrently, the expression level of mTORC1-related proteins in the “MBCD group” was the lowest. Furthermore, addition of cholesterol reversed the downregulation of the mTORC1 signaling pathway in “MBCD group” (Fig. 7B). This indicated that cholesterol mediates the up-regulation of the mTORC1 signaling pathway via LRP8.

Autophagy-related proteins were then analyzed. LC3B-II/I expression was higher and p62 expression lower in “untreated group” shLRP8 cells compared to NC controls. In shLRP8 AMO1 and NCI-H929 cell lines, compared with “untreated group”, the expression of LC3B II/I significantly decreased in “cholesterol group”, while the protein expression of P62 increased, suggesting that the addition of cholesterol could reverse the promoting effect of LRP8 knockdown on autophagy; the expression of LC3B II/I in “MBCD group” was significantly higher than that in “untreated group”, indicating that cholesterol depletion further enhanced cellular autophagy; the expression of LC3B II/I in “MBCD + cholesterol group” was significantly lower than that in “MBCD group”, and the protein expression of P62 increased, suggesting that the addition of cholesterol could reverse the promoting effect of MBCD on autophagy (Fig. 7C). Immunofluorescence further confirmed these results. Compared to NC cells, shLRP8 cells displayed an increase in autophagosomes and autolysosomes. Upon adding cholesterol to shLRP8 cells, the numbers of autophagosomes and autolysosomes decreased. When cholesterol was depleted by MBCD, the amounts of autophagosomes and autolysosomes increased. The cells under the “MBCD + cholesterol” treatment reversed the upregulation of autophagy observed in cells treated with MBCD (Fig. 7D). These findings suggest that cholesterol mediates the regulatory effect of LRP8 on autophagy.

We further subjected cells to MHY1485 (mTOR agonist) at a concentration of 10 μM for a duration of 4 hours. The outcomes of the WB assays manifested that in the shLRP8 group of cells upon the stimulation of MHY1485, the protein expression magnitudes of p-mTOR, p-p70s6k and p-4ebp exhibited a significant increase (Fig. 7E). This suggests that MHY1485 counteracts the inhibitory impact of LRP8 knockdown on the mTORC1. Furthermore, we found that after treating the cells with MHY1485, the expression level of LC3B-II/I decreased, the expression level of P62 increased (Fig. 7F), and the number of autophagosomes and autolysosomes decreased (Fig. 7G). This suggest that the treatment of cells with MHY1485 blocked autophagy, reversed the promoting effect of LRP8 knockdown on autophagy in cells.

### LRP8 knockdown-induced apoptosis could be aggravated by autophagy inhibitor 3-methyladenine in MM cells

**Fig. 8:**
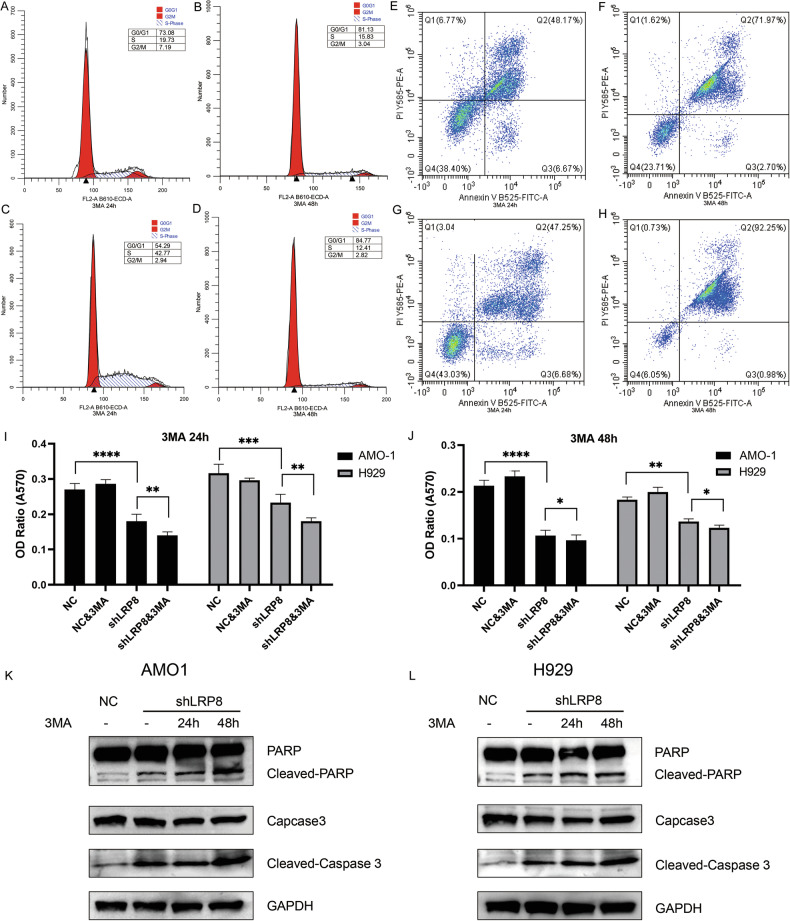
The decreased cholesterol uptake due to the suppression of LRP8 expression leads to an augmented autophagy level in MM cells, and this autophagy acts as a protective mechanism under the circumstances of nutrient deficiency. **A**, **B** Flow cytometry revealed that in the shLRP8 AMO1 cell line, 24 h (**A**)/48 h (**B**) treatment with 3MA led to a further increase in cell cycle arrest, and the proportion of cells in the G2/M phase was even lower than before 3MA addition. **C**, **D** Flow cytometry revealed that in the shLRP8 H929 cell line, 24-hour (**C**)/48-hour (**D**) treatment with 3MA led to a further increase in cell cycle arrest, and the proportion of cells in the G2/M phase was even lower than before 3MA addition. **E**, **F** Flow cytometry indicated that in the shLRP8 AMO1 cell line, exposure to 3MA for 24 h (**E**)/48 h (**F**) would cause a further elevation in the proportion of apoptotic cells. **G**, **H** Flow cytometry indicated that in the shLRP8 H929 cell line, exposure to 3MA for 24 h (**G**)/48 h (**H**) would cause a further elevation in the proportion of apoptotic cells. **I**, **J** Influence of 3-MA on the viability of AMO1 and H929 cell lines via MTT analysis. Upon the addition of autophagy inhibitors, the survival rate of MM cells decreased. **K**, **L** The alterations in the expression levels of apoptosis-related proteins in AMO1 (**K**) and H929 (**L**) cell lines treated with 3-MA were investigated. Compared with NC cells, following the downregulation of LRP8 in MM cells, cleaved caspase 3 and PARP were significantly upregulated. After the addition of 3-MA, the expression levels of cleaved caspase 3 and PARP were further elevated).

To further clarify whether the enhanced autophagy induced by LRP8 knockout in MM cells promotes or inhibits apoptosis, we utilized the autophagy inhibitor 3-methyladenine (3MA) to investigate its impact on MM cell. Flow cytometry revealed that 3-MA treatment (24/48 hours) further suppressed the cell cycle and increased apoptosis in MM cells (Fig. 8A–H). MTT assays demonstrated significantly reduced cell survival upon 3-MA addition (Fig. 8I, J). Then, we explored the alterations of apoptosis-related proteins. Western blot analysis showed that LRP8 knockdown upregulated cleaved caspase-3 and cleaved PARP (Fig. 8K, L). Moreover, when autophagy was inhibited, cleaved caspase 3 and cleaved PARP were further enhanced. These findings suggest that the elevated autophagy level resulting from the downregulation of LRP8 acts as a protective mechanism employed by MM cells under the condition of cholesterol deficiency.

### Pan-cancer dependency analysis of LRP8

To further observe whether LRP8 possesses a distinctive prognostic value solely in myeloma, we conducted a pan-cancer dependency analysis of LRP8 via the DepMap database and calculated the dependency scores of LRP8 across different cancer lineages. Through evaluating the CRISPR-Cas9 gene effect analysis (CERES score) in DepMap, we discovered that LRP8 exhibited an extremely high dependency in lymphoid and myeloid tumor cells. Specifically, the highest dependency was observed in mature B-cell lymphoma, which once again accentuates the significant value of LRP8 in myeloma. Secondly, the second-highest LRP8 dependency score was found in B-cell lymphoma. In T-cell lymphoma, LRP8 also manifested a high dependency, although it was marginally lower than that in B-cell lymphoma. Among solid tumors, some solid tumors displayed varying degrees of dependency. LRP8 demonstrated a relatively high dependency in colon cancer and ovarian cancer, and also exhibited a certain degree of dependency in central nervous system tumors and pancreatic tumors. We presented the dependency scores of different tumors on LRP8 in Fig.S1 (Fig.S1A). We also displayed the expression of LRP8 in different tumor cell lines (Fig.S1B). Subsequently, we further explored the prognostic value of LRP8 for different tumors. We analyzed the influence of LRP8 on patient OS in the TCGA and GTEx databases. We found that LRP8 had significant prognostic value in mixed kidney cancer, papillary renal cell carcinoma, clear cell renal cell carcinoma, and acute myeloid leukemia, etc. (Fig.S2).

## Discussion

Here, we determined the significance and prognostic value of cholesterol metabolism for MM patients through large-scale samples. We confirmed that the reduction of serum cholesterol was related to the tumor burden and cholesterol uptake in MM. Through further exploration, we discovered the important role and prognostic value of LRP8 in the cholesterol metabolism of MM cells. Our research revealed that LRP8, by enhancing the utilization and uptake of cholesterol in MM cells, anomalously activates the cholesterol-related mTORC1 pathway within MM cells, lowers the autophagy level, and promotes the proliferation and cellular activity of MM cells. Inhibiting LRP8 leads to a decrease in cholesterol uptake by MM cells, augments cell apoptosis, but induces the occurrence of protective autophagy in the cells.

Cellular metabolism has always been a research hotspot in the field of tumors [18]. In MM, although we frequently discover the prognostic value of cholesterol and also confirm the inhibitory function of statins on the proliferation of MM cells, it has not received sufficient attention. The proliferation, survival and biological activity of MM tumor cells, coupled with the continuous secretion of immunoglobulins and light chains by MM cells, confer greater significance on cell metabolism in MM. Our study is the first to deeply explore the significance and key regulators of cholesterol metabolism in MM and explain the inherent meaning and mechanism of the decreased serum cholesterol levels in MM patients.

The expression level of LRP8 is significantly elevated in types of tumors [19–21]. In breast cancer, LRP8 could promote the proliferation and migration of tumor cells by activating the Wnt/β-Catenin pathway [6, 22]. In addition, LRP8 can induce ERK1/2 phosphorylation and activate the cell cycle [23].

Our research has confirmed that in MM, LRP8 activates the mTORC1 pathway through a cholesterol-dependent route. The mTORC1 pathway is one of the most important pathways in the pathogenesis of MM [24, 25]. We have found that LRP8 activates the mTORC1 pathway by highly uptaking cholesterol in MM and further downregulates the autophagy level of MM cells in the context of abundant cholesterol supply. mTORC1, as an important autophagy regulator [26], undergoes multiple metabolism-related changes after receiving the cholesterol signal transmitted by LRP8.

Different from the definite oncogenic role of the mTORC1 in MM, the influence of autophagy presenting a dual role [27]. In our research, regarding the LRP8-mTORC1-autophagy response, the alterations of mTORC1 and autophagy constitute the overall metabolic changes in MM cells following the uptake of cholesterol via LRP8. After the expression level of LRP8 is upregulated and the uptake of cholesterol by MM cells increases, the autophagy level of the cells declines. We suggest that this might be a form of energy metabolism regulation under a state of sufficient nutrition. When the expression level of LRP8 is attenuated, the cholesterol-uptaking capacity of MM cells is compromised, the mTORC1 pathway is repressed, facilitating the apoptotic process of MM cells and engendering an augmented level of protective autophagy within MM cells. Further repression of autophagy can elicit a more conspicuous apoptotic phenomenon in MM cells. Our discoveries have verified the paramount role of cholesterol metabolism in MM and insinuate that LRP8 may potentially serve as a metabolic target for the therapeutics of multiple myeloma.

## Methods and Materials

### Patient characteristics

The clinical and clinicopathological data of patients were derived from a cohort of 703 newly diagnosed multiple myeloma (MM) patients between 2014 and 2021. These patients received first-line treatments primarily involving proteasome inhibitors and/or immunomodulatory drugs, with regimens including at least two therapeutic agents.

For the analysis of LRP8 expression levels and prognosis, bone marrow samples were collected from 108 newly diagnosed MM patients between 2016 and 2020. Only patients who had not undergone autologous hematopoietic stem cell transplantation (ASCT) were included. The initial treatment regimen for these patients consisted of a three-drug combination: a protease inhibitor, lenalidomide, and dexamethasone. Bone marrow samples were isolated using CD138 magnetic beads for subsequent analyses.

### Single cell mRNA sequencing

Bone marrow samples were obtained from Zhongshan Hospital, Fudan University. Mononuclear cells were isolated and used for single-cell RNA sequencing (scRNAseq) using Oebiotech’s services in China. Single-cell libraries were generated from unsorted bone marrow mononuclear cells (BMMCs) using the 10× Genomics Chromium Single Cell 5′ Library Kit and sequenced on an Illumina platform. FASTQ files were aligned to the GRCh38 reference genome using Cell Ranger (v5.0.1). Seurat v4.0.1 was employed for clustering and dimensionality reduction. Pathway activation analysis was conducted using the irGSEA package and AUCell method based on gene sets from the MSigDB database.

### Bulk RNA sequencing

Cells were processed into granules, and RNA was extracted for library preparation using Oebiotech’s services. Transcriptomic RNA sequencing was performed on the Illumina NovaSeq 6000 platform, generating paired-end reads of 150 bp length. Differentially expressed genes were identified using the limma software, and Gene Set Enrichment Analysis (GSEA) [28] was conducted on these genes.

### Cell culture

The AMO1 and H929 cell lines were sourced from the Institute of Basic Medical Sciences, Chinese Academy of Medical Sciences. Cells were cultured in RPMI 1640 medium supplemented with 10% fetal bovine serum (FBS) under conditions of 37 °C, saturated humidity, and 5% CO_2_.

### Lentiviral gene transduction

LRP8-overexpression and LRP8-knockdown lentiviruses were constructed using the Ubi-MCS-3FLAG-CBh-gcGFP-IRES-puromycin vector (Hanbio, China). Based on viral titer and MOI, the appropriate volume of lentivirus was added to the cell suspension. Stable transfectants were selected by adding puromycin to achieve a final concentration of 5 μg/mL. Successfully transformed cell lines were prepared for further experiments.

### Flow cytometry

1 × 10^6/ cells were treated with 5 mL of cold ethanol at −20 °C overnight for fixation. The cells were resuspended in PBS, supplemented with a 10 μl RNase A stock solution, and incubated at room temperature for 1 h. Subsequently, the cells were stained with 40 μg/mL propidium iodide (PI) solution, and the cell cycle was analyzed using a Flowsight flow cytometer (Merck Millipore, Germany). For the cell apoptosis experiment, cells treated with 2 μg/mL for 48 h at a density of 2 × 10^5 cells were collected. The collected cells were then stained under dark conditions using Annexin-V-Allophycocyanin (BioLegend, USA) and PI for 15 min. Finally, the apoptosis of the cells was detected using a flow cytometer.

### Quantitative RT-PCR analysis

RNA was extracted from cells using the EZ Bioscience RNA extraction kit according to the manufacturer’s protocol. cDNA was synthesized using the Takara cDNA Synthesis Kit. Real-time PCR was performed using the Takara TB Green Premix Ex Taq (Tli RNaseH Plus) quantitative PCR kit. Primer sequences are provided in Supplementary Table 3. Data were normalized to means with standard deviations.

### Western blotting

Cells were lysed in RIPA buffer (Thermo Fisher, USA) containing a protease inhibitor cocktail (Yeasen, China). Protein extracts were subjected to SDS-PAGE electrophoresis and transferred onto PVDF membranes (Millipore, Germany). Membranes were blocked with 5% skimmed milk and incubated with primary antibodies overnight at 4 °C. Detection was performed using HRP-conjugated secondary antibodies and visualized with Super ECL detection reagent (ECL BioSharp, China).

### Transwell migration and invasion assays

Transwell chamber with 8.0 μm pore size was used to measure cell migration, and Matrigel transwell chamber was used for cell invasion tests (Corning, USA). Cells were initially inoculated onto top-perforated membranes and incubated for 24 h (migration experiment) or 48 h (invasion experiment). After incubation, cells in the upper part of the insert were removed and cells adhering to the lower part were fixed, stained with crystal violet, photographed at three locations, and counted using Image J (NIH, US).

### MTT assays

Cells were quantified by Trypan blue staining and a hemocytometer and subsequently seeded into 96-well plates at a density ranging from 3000 to 4000 cells per well. After the treatments, the cells were subjected to MTT for 4 h. Subsequently, the supernatant was withdrawn and the formazan crystals were dissolved in 200 μL of dimethyl sulfoxide (DMSO). Finally, the optical density was determined at 570 nm.

### Immunohistochemistry (IHC) analysis

Mouse xenograft tumor tissues were paraffin-embedded and sectioned. IHC was performed following the manufacturer’s protocol for the IHC kit (Sevier, China). Primary antibodies are listed in Supplementary Table 4.

### Detection of autophagy flow

The mRFP-GFP-LC3 adenovirus (Wanleibio, China) was utilized for real-time surveillance of autophagy. Target cells were infected with lentivirus. The cells were gathered through centrifugation and counted. Five hundred thousand cells were inoculated into each well of the 6-well plates (1 ml/well), with the virus added at a multiplicity of infection (MOI) of 20. Six hours after infection, the cells were re-collected by centrifugation, the supernatant was removed, the cells were re-suspended in fresh medium, and re-planted back into the corresponding wells for continuous culture. 48 hours following virus infection, the cells were obtained via centrifugation. The autophagy state of the cells was observed and captured by a laser confocal microscope (1000X).

### Subcutaneous xenograft tumor mouse model

During logarithmic growth phase, cells were harvested, washed with PBS, and resuspended to a density of approximately 5 × 10^6 cells/100 μL. These cells were subcutaneously injected into B-NDG mice. Five randomly assigned 5-week-old female mice were divided into control (NC) and LRP8-overexpression (LRP8-OE) groups.

### Xenograft tumor mouse model through intravenous injection

Cells were harvested during logarithmic growth, washed with PBS, and resuspended at a density of 10^7 cells/100 μL. Within 2.5 h, cells were intravenously injected into the tail veins of B-NDG mice. Each group of five 5-week-old female mice was randomly assigned to either the NC or LRP8-OE group. Tumor development was monitored consistently.

### Image acquisition and quantitative analysis

All Western blot membranes were imaged using a chemiluminescence imaging system under non-saturating exposure conditions. Protein band quantification was conducted using ImageJ software (version 1.54k). Specifically, regions of interest (ROIs) were manually outlined around the target bands, followed by background subtraction using the “Rolling Ball” algorithm with a radius of 50 pixels. Integrated optical density (IOD) values were normalized to corresponding loading controls (e.g., β-actin or GAPDH) to account for potential variations in protein loading and transfer efficiency. Prior to hypothesis testing, normality was verified using the Shapiro-Wilk test, and homogeneity of variance was assessed via Levene’s test. Intergroup differences were analyzed using two-tailed unpaired Student’s t-tests performed in GraphPad Prism version 8.0 (GraphPad Software). Statistical significance was defined as follows: *p < 0.05, **p < 0.01, ***p < 0.001, and ****p < 0.0001. The outcomes are presented in Fig.S3.

### Statistical analysis

Statistical analyses were conducted using R version 4.0.3 and Stata statistical software, version 14.0. Two-sided *P*-values were used with a significance threshold of *P* < 0.05. Data were presented as means ± SD and analyzed using GraphPad Prism. Group differences were evaluated using Student’s t-test or Wilcoxon test, while survival data were visualized with Kaplan–Meier curves and analyzed via log-rank tests.

## Supplementary information

Supplementary Table 1

Supplementary Table 2

Supplementary Table 3

Supplementary Table 4

Supplementary Figure S1

Supplementary Figure S2

Supplementary Figure S3

Original Data File

## Data Availability

The single-cell RNA-seq processed gene expression data reported in this paper have been deposited into the CNGB Sequence Archive (CNSA) [29] of the China National GeneBank DataBase (CNGBdb) [30] with accession number CNP0005613(CSE0000446), and raw sequencing data have been deposited in the OMIX, China National Center for Bioinformation, Chinese Academy of Sciences (accession no. OMIX007547) [31]. To request access to raw sequencing data, please apply at the Human Genetic Resources Service System of the Ministry of Science and Technology (http://apply.hgrg.net/) in accordance with the Regulations on the Management of Human Genetic Resources of China. The multiple myeloma RNA-seq data GSE24080 were downloaded from the GEO database. Analysis code described in the manuscript will be made available upon request to the corresponding author pending approval.
