# Supplementary material for: LRP8-dependent cholesterol metabolism modulates mTORC1 signaling and apoptotic pathways in multiple myeloma
Source: Cell Death Dis. 2025 Apr 8;16(1):263. doi: 10.1038/s41419-025-07625-w (PMC11978852; doi:10.1038/s41419-025-07625-w)
Supplement: Supplementary file 1 — Supplementary Table 1 [file 41419_2025_7625_MOESM1_ESM.docx]

**Supplementary Table 1. Baseline characteristics of patients in different cohort with multiple myeloma.**

| Variable | Fudan | TT2 | TT3 | MMRF |
| --- | --- | --- | --- | --- |
| Patinets(No) | 703 | 345 | 214 | 785 |
| Gender(No,%) |  |  |  |  |
| Male | 442(62.9) | 197(57.1) | 140(65.4) | 464(59.1) |
| Female | 261(37.1) | 148(42.9) | 74(34.6) | 321(40.9) |
| Age(year, (No,%)) |  |  |  |  |
| <60 | 165(23.5) | 212(61.4) | 106(49.5) | 262(33.4) |
| ≥60 | 538(76.5) | 133(38.5) | 108(50.5) | 489(62.9) |
| NA | 0(0.0) |  | 0(0.0) | 34(4.3) |
| ISS stage(No,%) |  |  |  |  |
| I | 191(27.2) | 190(55.1) | 107(50.0) | 265(33.8) |
| II | 188(26.7) | 82(23.8) | 61(28.5) | 277(35.3) |
| III | 324(46.1) | 73(21.1) | 46(21.5) | 221(28.1) |
| NA | 0(0.0) | 0(0.0) | 0(0.0) | 22(2.8) |
| FISH high-risk(No,%) |  |  |  |  |
| Detected | 354(50.4) | 132(38.3) | 75(35.0) | NA |
| Not detected | 349(49.6) | 213(61.7) | 139(65.0) | NA |
| Follow-up time (month, (median,range)) | 23.6(0.1-96.8) | 59.9(0.0-98.5) | 39.2(0.77-52.5) | 26.4(0.0-66.1) |
| Progress(No,%) | 315(44.8) | 194(56.2) | 55(25.7) | NA |
| Dead(No,%) | 190(27.0) | 129(37.4) | 43(20.1) | 162(20.6) |
