# Supplementary material for: LRP8-dependent cholesterol metabolism modulates mTORC1 signaling and apoptotic pathways in multiple myeloma
Source: Cell Death Dis. 2025 Apr 8;16(1):263. doi: 10.1038/s41419-025-07625-w (PMC11978852; doi:10.1038/s41419-025-07625-w)
Supplement: Supplementary file 2 — Supplementary Table 2 [file 41419_2025_7625_MOESM2_ESM.docx]

**Supplementary Table 2. Baseline characteristics of 108 patients with multiple myeloma.**

| Variable | Total | LRP8_High | LRP8_Low |
| --- | --- | --- | --- |
| Patinets(No) | 108 | 54 | 54 |
| Gender(No,%) |  |  |  |
| Male | 74(68.5) | 41(75.9) | 33(61.1) |
| Female | 34(31.5) | 13(24.1) | 21(38.9) |
| Age(year, (No,%)) |  |  |  |
| <60 | 30(27.8) | 15(27.8) | 11(20.4) |
| ≥60 | 78(72.2) | 39(72.2) | 43(79.6) |
| ISS stage(No,%) |  |  |  |
| I | 43(39.8) | 12(22.2) | 26(48.1) |
| II | 28(25.9) | 13(24.1) | 14(25.9) |
| III | 37(34.3) | 29(53.7) | 14(25.9) |
| FISH high-risk(No,%) |  |  |  |
| Detected | 34(31.5) | 26(48.1) | 22(40.7) |
| Not detected | 74(68.5) | 28(51.9) | 32(59.3) |
| Follow-up time (month, (median,range)) | 48.7(2.1-103.0) | 44.9(2.1-103.0) | 51.3(5.4-77.6) |
| Progress(No,%) | 53(49.1) | 32(59.3) | 21(38.9) |
| Dead(No,%) | 34(31.5) | 24(44.4) | 10(18.5) |
