# Supplementary material for: LRP8-dependent cholesterol metabolism modulates mTORC1 signaling and apoptotic pathways in multiple myeloma
Source: Cell Death Dis. 2025 Apr 8;16(1):263. doi: 10.1038/s41419-025-07625-w (PMC11978852; doi:10.1038/s41419-025-07625-w)
Supplement: Supplementary file 3 — Supplementary Table 3 [file 41419_2025_7625_MOESM3_ESM.docx]

**Supplementary Table 3. Primer sequences used in the study.**

| Gene | Forward（5’→3’） | Reverse (5’→3’) |
| --- | --- | --- |
| GAPDH | GAAGGTGAAGGTCGGAGTC | GAAGATGGTGATGGGATTTC |
| LRP8 | CCTGCGAGGGTTCATGTATT | GGCTCAGGAAGTCAGTGGAG |
| shLRP8 | CCGGGACCTCAAGATTGGCTTTGAACTCGAGTTCAAAGCCAATCTTGAGGTCTTTTT | AATTAAAAAGACCTCAAGATTGGCTTTGAACTCGAGTTCAAAGCCAATCTTGAGGTC |
