# Supplementary material for: LRP8-dependent cholesterol metabolism modulates mTORC1 signaling and apoptotic pathways in multiple myeloma
Source: Cell Death Dis. 2025 Apr 8;16(1):263. doi: 10.1038/s41419-025-07625-w (PMC11978852; doi:10.1038/s41419-025-07625-w)
Supplement: Supplementary file 4 — Supplementary Table 4 [file 41419_2025_7625_MOESM4_ESM.docx]

**Supplementary Table 4. Antibodies used in Western blot, IHC and ELISA, along with pharmacological agents for cellular treatment.**

| Reagent | Company | Catalog number |
| --- | --- | --- |
| Anti-LRP8 Antibody | Abmart, China | T57023 |
| Anti- GAPDH Antibody | Proteintech, China | 60004-1-Ig |
| Anti-mTOR Antibody | Abcam, UK | ab134903 |
| Anti-p-mTOR Antibody | Abmart, China | T56571 |
| Anti-p70S6K Antibody | Abcam, UK | ab32529 |
| Anti-p-p70S6K Antibody | Abmart, China | T55261 |
| Anti-4EBP Antibody | Abcam, UK | ab32024 |
| Anti-p-4EBP Antibody | Abmart, China | T59231 |
| Anti-p62 Antibody | Abmart, China | T55546 |
| Anti-LC3B Antibody | Abmart, China | T55992 |
| Anti-Caspase3 Antibody | Abmart, China | T40044 |
| Anti-PARP Antibody | Abmart, China | T40050 |
| Anti-rabbit IgG，HRP-linked Antibody | Jackson, USA | AB_2338015 |
| Anti-mouse IgG, HRP-linked Antibody | Jackson, USA | AB_2338728 |
| Cholesterol Elisa Kit | YOBIBIO, China | U96-1290E |
| IgA Elisa Kit | YOBIBIO, China | U96-3215E |
| MBCD | MCE, USA | HY-101461 |
| Cholesterol | MCE, USA | HY-N0322 |
| MHY1485 | MCE, USA | HY-B0795 |
| 3MA | MCE, USA | HY-19312 |
