# Supplementary figures and images for: LRP8-dependent cholesterol metabolism modulates mTORC1 signaling and apoptotic pathways in multiple myeloma
Source: Cell Death Dis. 2025 Apr 8;16(1):263. doi: 10.1038/s41419-025-07625-w (PMC11978852; doi:10.1038/s41419-025-07625-w)

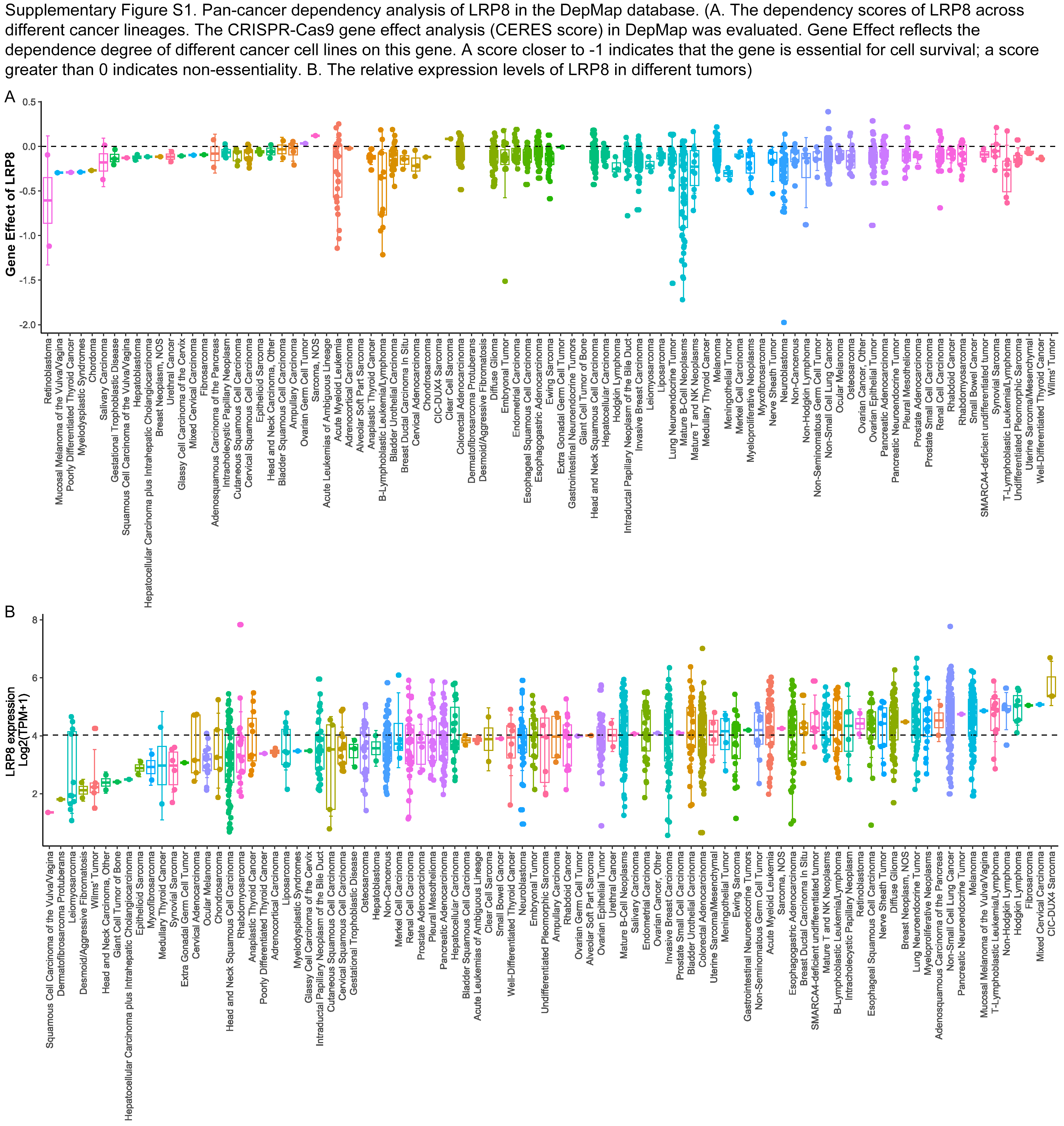

Supplement: Supplementary file 5 — Supplementary Figure S1 [file 41419_2025_7625_MOESM5_ESM.tif]

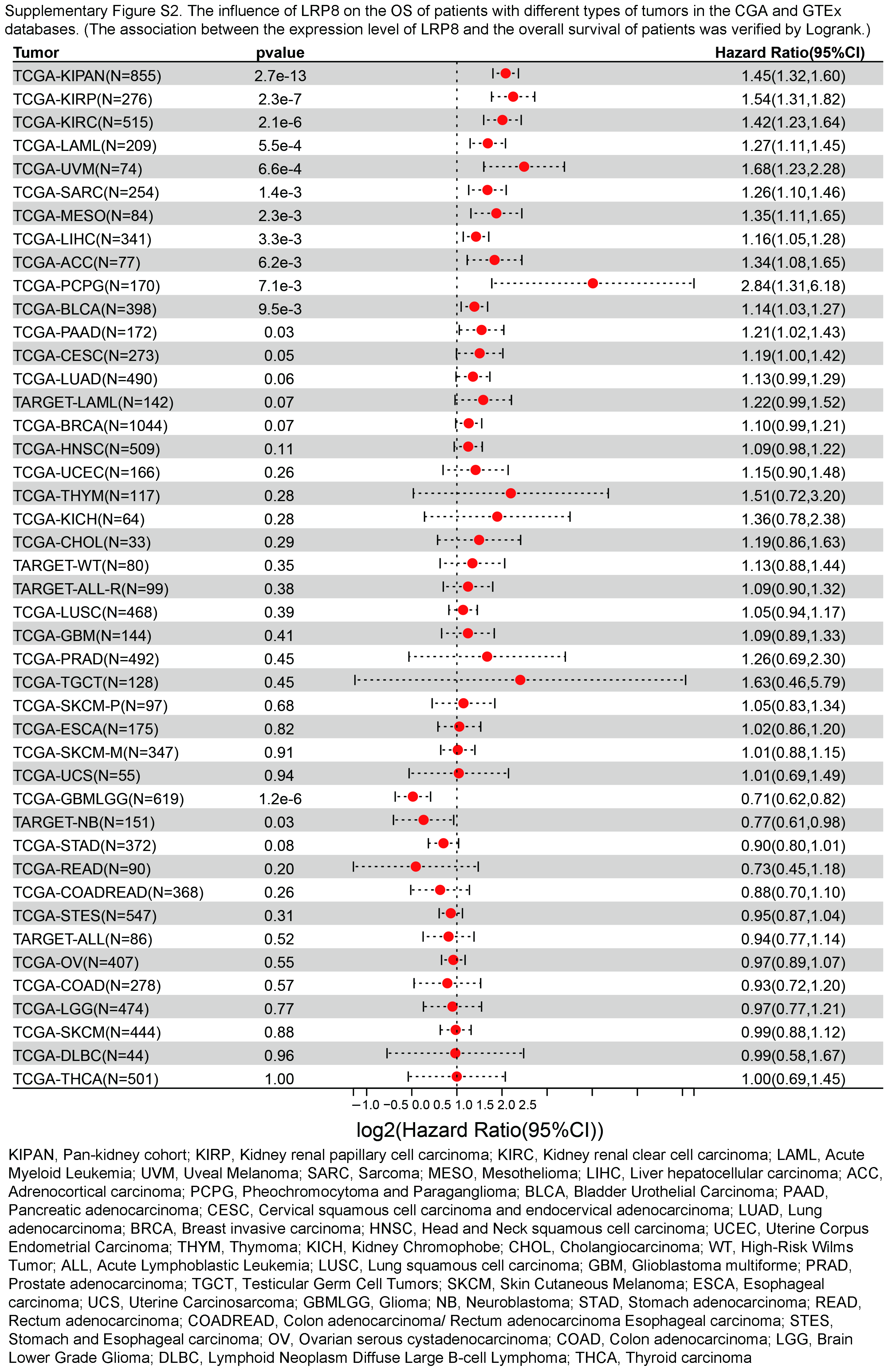

Supplement: Supplementary file 6 — Supplementary Figure S2 [file 41419_2025_7625_MOESM6_ESM.tif]
