# Supplementary material for: LRP8-dependent cholesterol metabolism modulates mTORC1 signaling and apoptotic pathways in multiple myeloma
Source: Cell Death Dis. 2025 Apr 8;16(1):263. doi: 10.1038/s41419-025-07625-w (PMC11978852; doi:10.1038/s41419-025-07625-w)
Supplement: Supplementary file 7 — Supplementary Figure S3 [file 41419_2025_7625_MOESM7_ESM.pdf]

Supplementary Figure S3. Analysis of Gray Values of Western Blot for Protein Expression of Different Cell Groups. (Analyzed by ImageJ and differences between groups evaluated by t-tests)

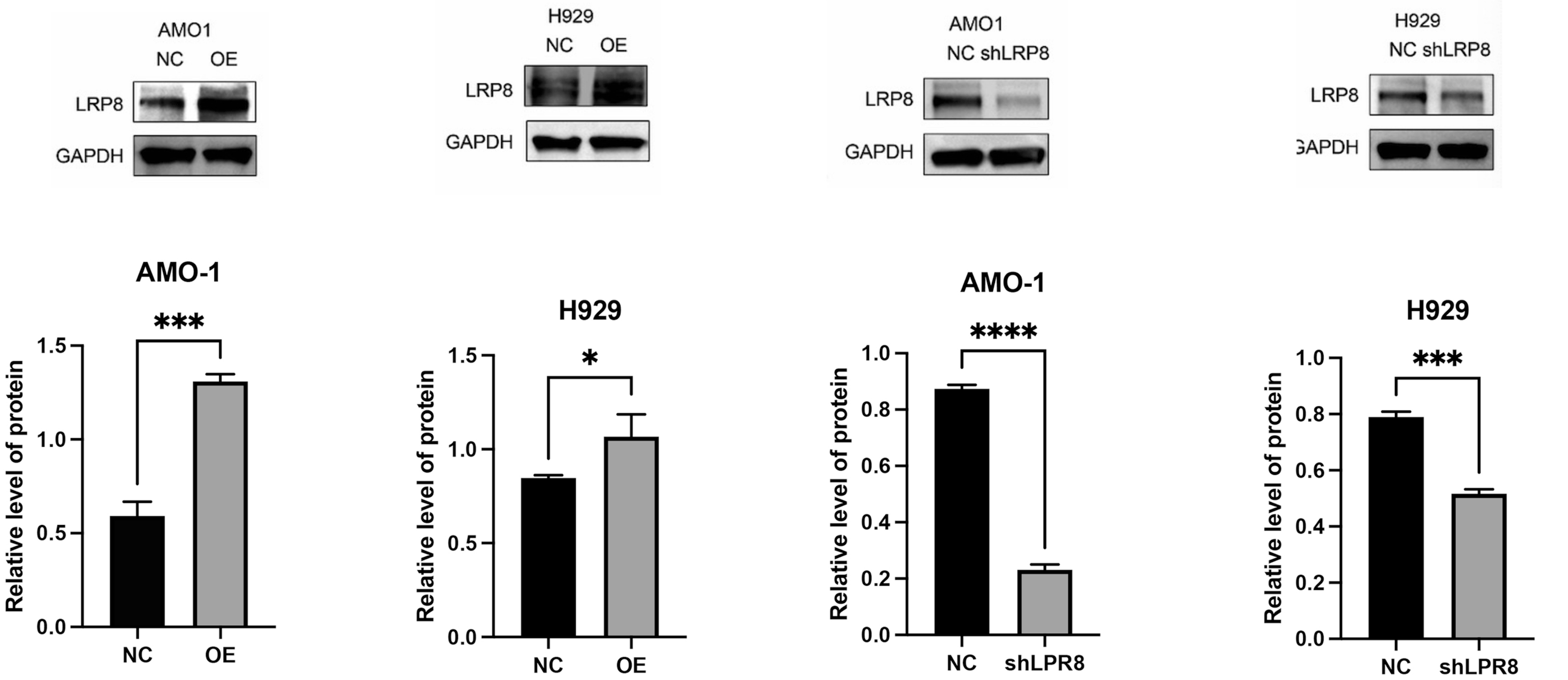

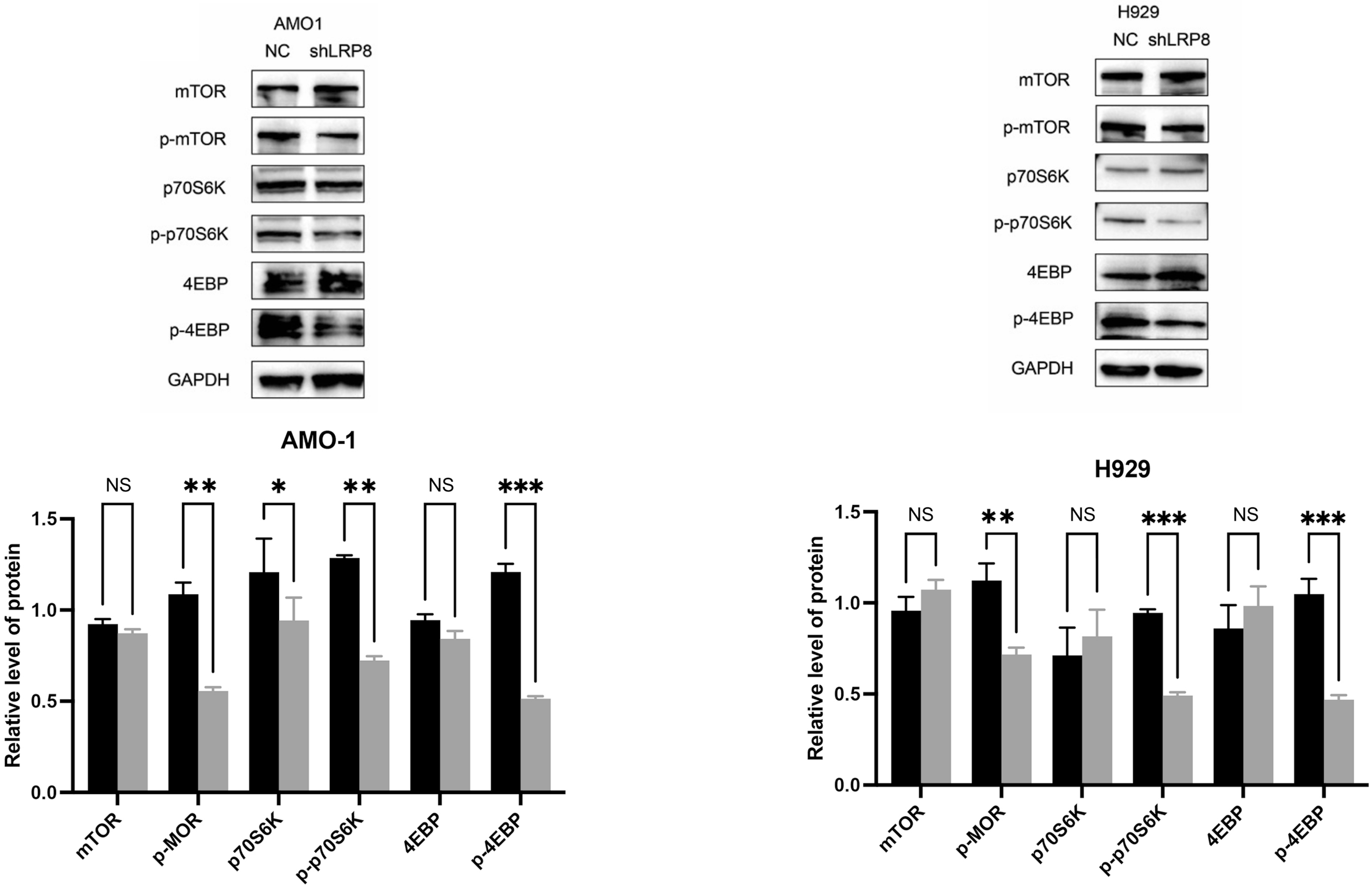
AMO1
NC
shLRP8
mTOR
p-mTOR
p70S6K
p-p70S6K
4EBP
p-4EBP
GAPDH
H929
NC
shLRP8
mTOR
p-mTOR
p70S6K
p-p70S6K
4EBP
p-4EBP
GAPDH
AMO-1
NS
**
*
**
NS
***
Relative level of protein
1.5
1.0
0.5
0.0
mTOR
p-MOR
p70S6K
p-p70S6K
4EBP
p-4EBP
H929
NS
**
NS
***
NS
***
Relative level of protein
1.5
1.0
0.5
0.0
mTOR
p-MOR
p70S6K
p-p70S6K
4EBP
p-4EBP

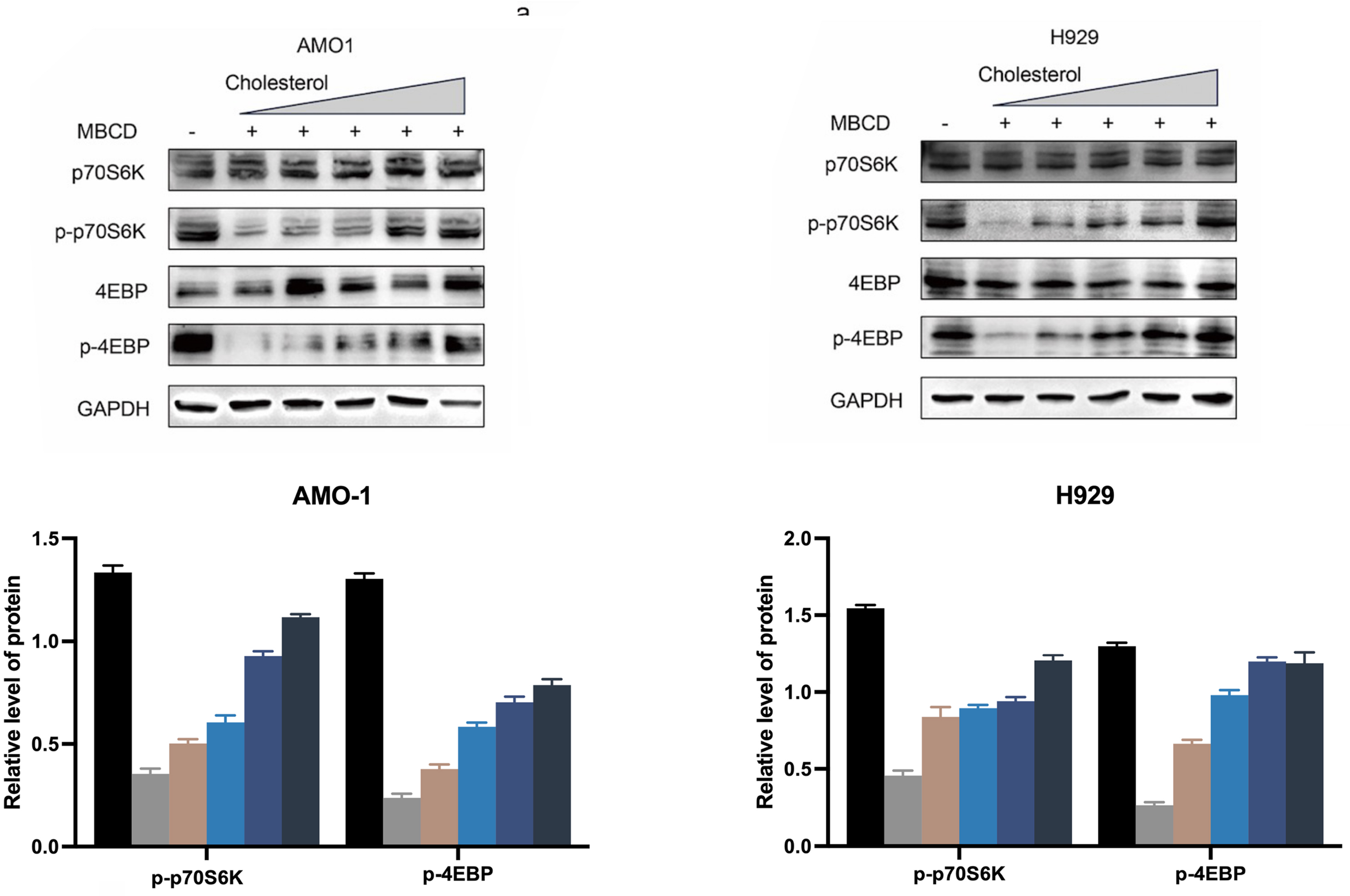
AMO1
Cholesterol
MBCD
- + + + + +
p70S6K
p-p70S6K
4EBP
p-4EBP
GAPDH
H929
Cholesterol
MBCD
- + + + + +
p70S6K
p-p70S6K
4EBP
p-4EBP
GAPDH
AMO-1
Relative level of protein
0.0
0.5
1.0
1.5
p-p70S6K
p-4EBP
H929
Relative level of protein
0.0
0.5
1.0
1.5
2.0
p-p70S6K
p-4EBP

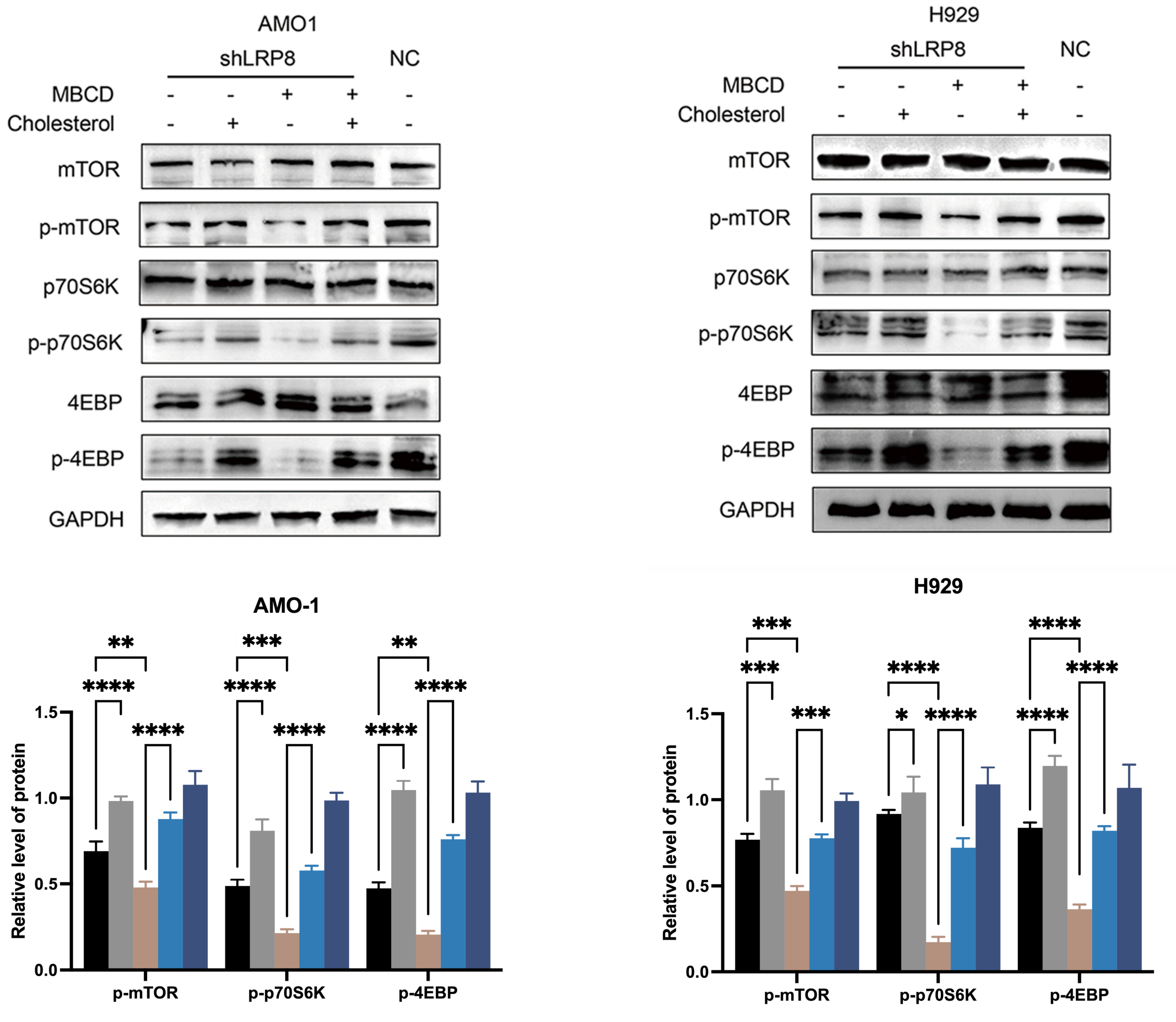

AMO1
shLRP8
NC
MBCD
Cholesterol
mTOR
p-mTOR
p70S6K
p-p70S6K
4EBP
p-4EBP
GAPDH
H929
shLRP8
NC
MBCD
Cholesterol
mTOR
p-mTOR
p70S6K
p-p70S6K
4EBP
p-4EBP
GAPDH
AMO-1
Relative level of protein
0.0
0.5
1.0
1.5
p-mTOR
p-p70S6K
p-4EBP
H929
Relative level of protein
0.0
0.5
1.0
1.5
p-mTOR
p-p70S6K
p-4EBP

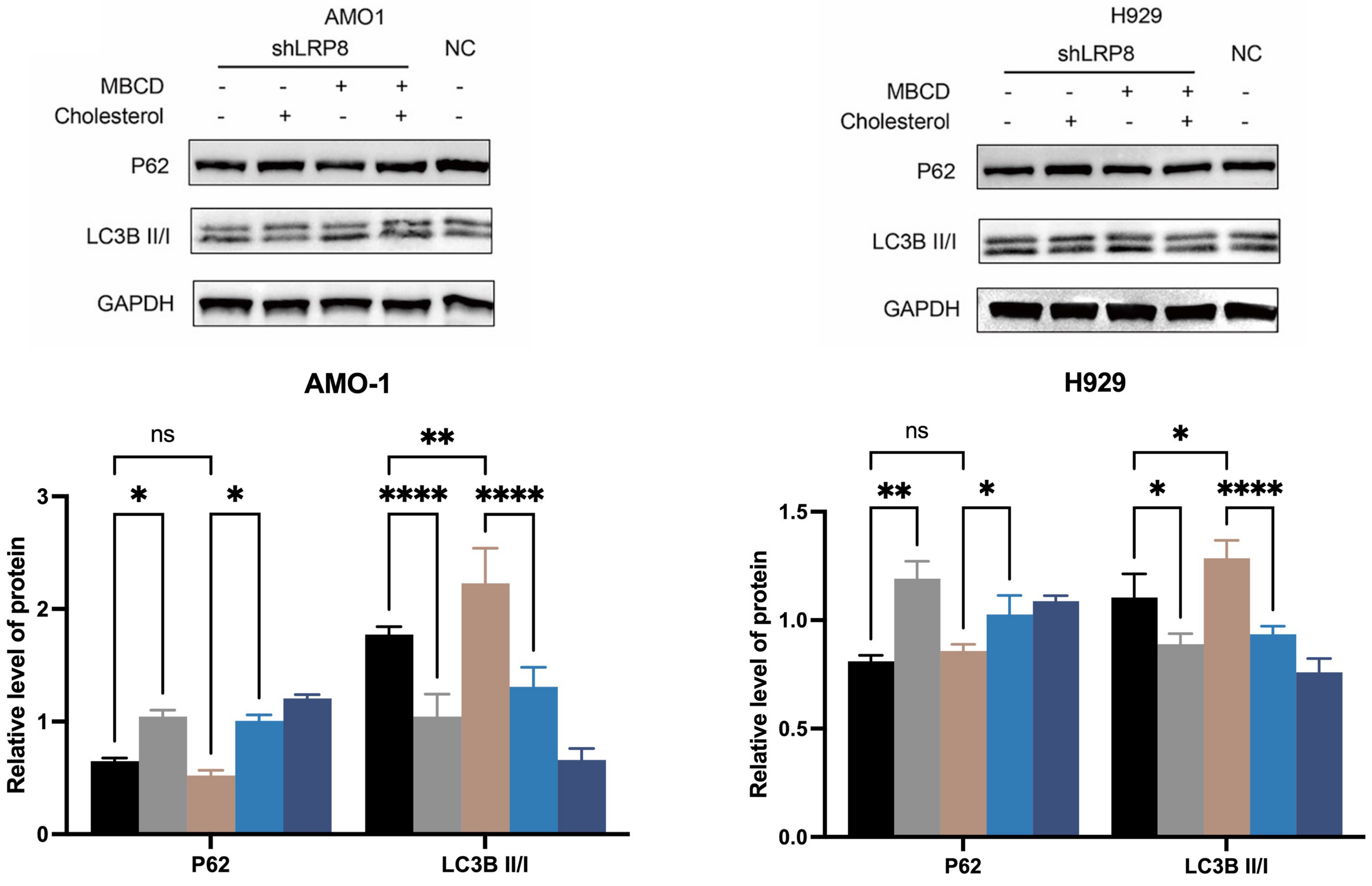
AMO1
shLRP8
NC
MBCD
Cholesterol
P62
LC3B II/I
GAPDH
H929
shLRP8
NC
MBCD
Cholesterol
P62
LC3B II/I
GAPDH
AMO-1
Relative level of protein
ns
**
*
*
****
****
P62
LC3B II/I
H929
Relative level of protein
ns
*
**
*
*
****
P62
LC3B II/I

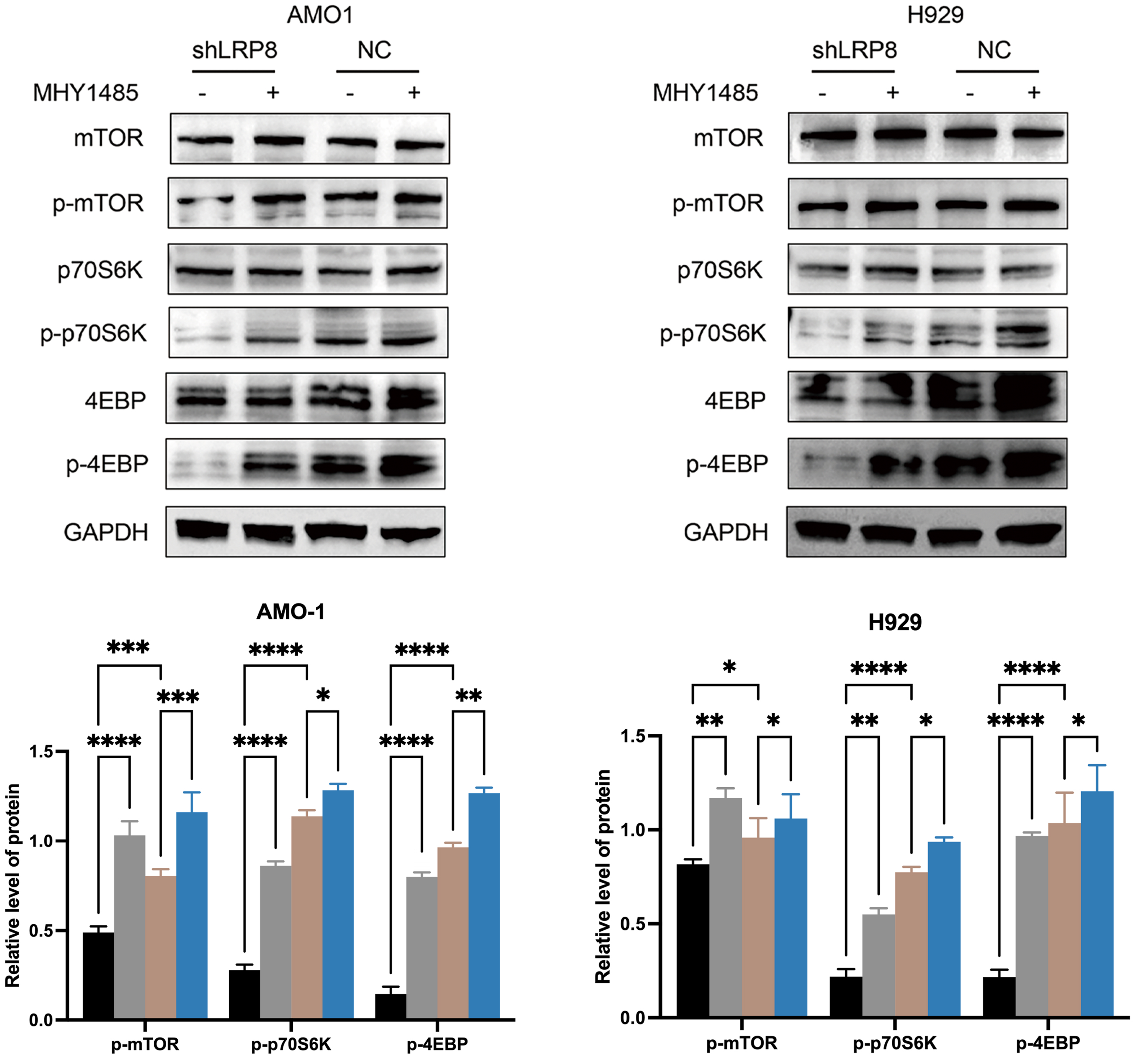
AMO1
shLRP8
NC
MHY1485
-
+
-
+
mTOR
p-mTOR
p70S6K
p-p70S6K
4EBP
p-4EBP
GAPDH
H929
AMO-1
Relative level of protein
p-mTOR
p-p70S6K
p-4EBP
***
****
*
**

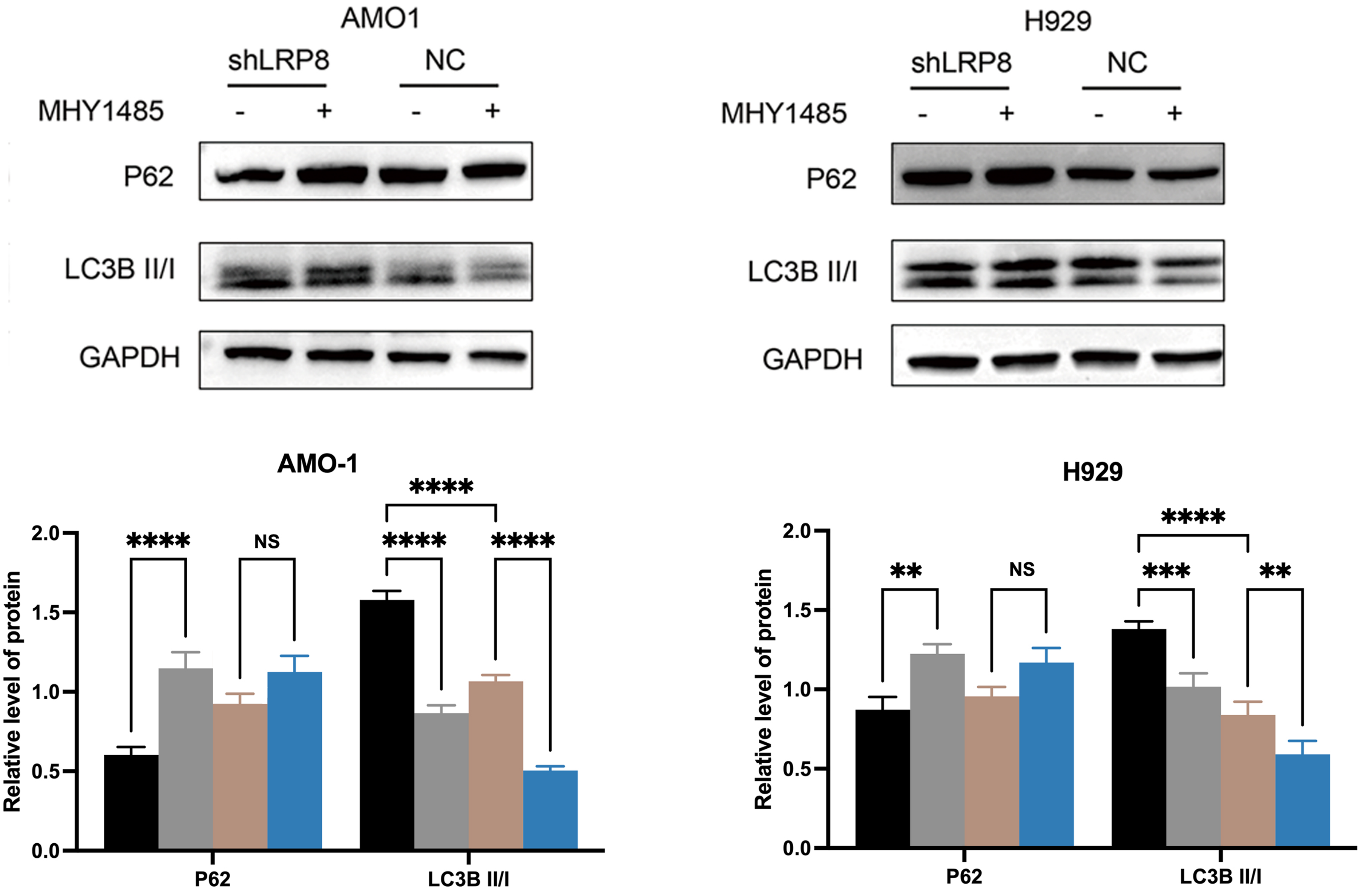

AMO1
shLRP8
NC
MHY1485
-
+
-
+
P62
LC3B II/I
GAPDH
H929
shLRP8
NC
MHY1485
-
+
-
+
P62
LC3B II/I
GAPDH
AMO-1
Relative level of protein
2.0
1.5
1.0
0.5
0.0
****
NS
****
****
****
P62
LC3B II/I
H929
Relative level of protein
2.0
1.5
1.0
0.5
0.0
**
NS
****
***
**
P62
LC3B II/I

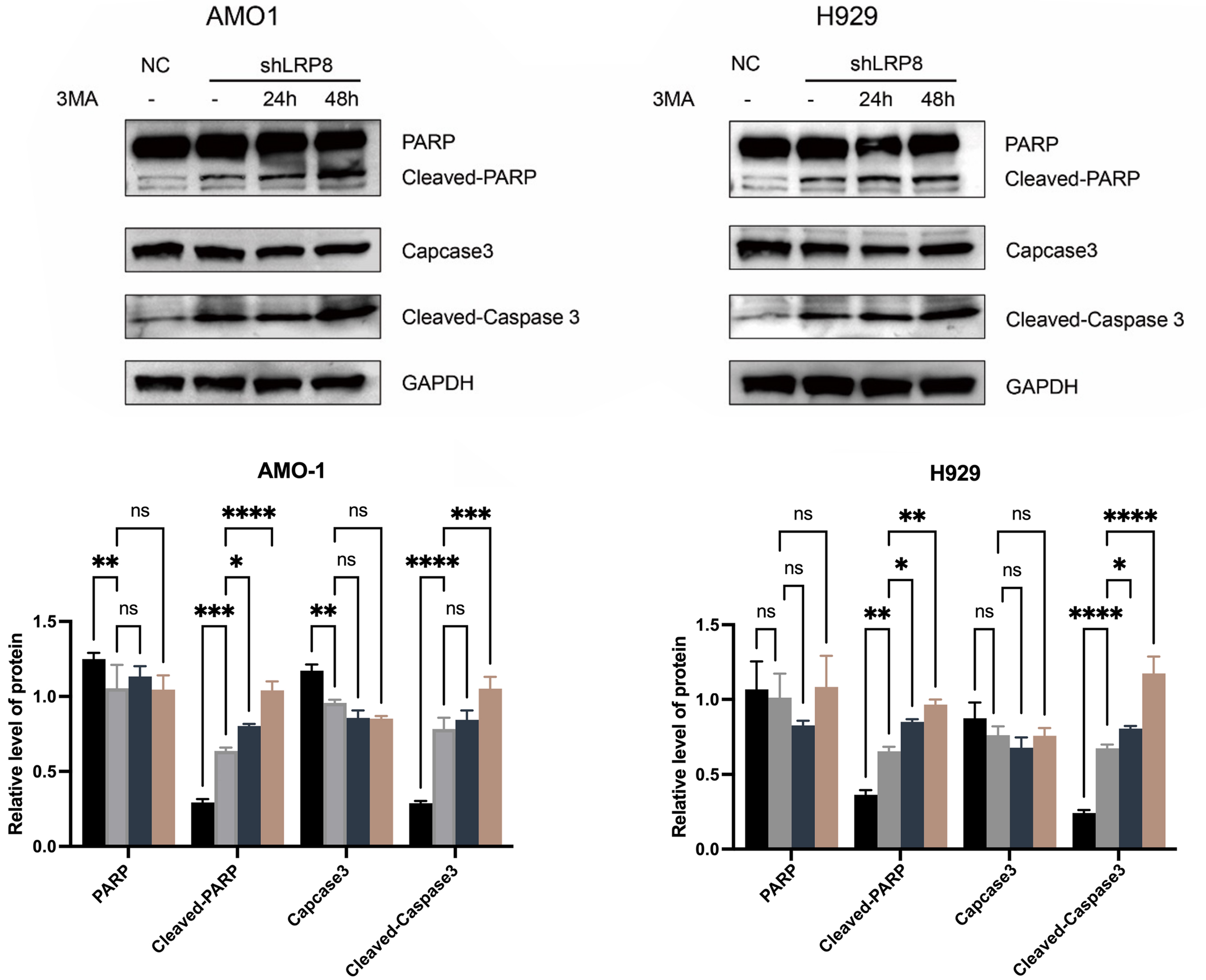
AMO1
NC
shLRP8
3MA
-
-
24h
48h
PARP
Cleaved-PARP
Capcase3
Cleaved-Caspase 3
GAPDH
H929
NC
shLRP8
3MA
-
-
24h
48h
PARP
Cleaved-PARP
Capcase3
Cleaved-Caspase 3
GAPDH
AMO-1
Relative level of protein
0.0
0.5
1.0
1.5
PARP
Cleaved-PARP
Capcase3
Cleaved-Caspase3
**
ns
ns
***
*
****
**
ns
ns
****
ns
***
H929
Relative level of protein
0.0
0.5
1.0
1.5
PARP
Cleaved-PARP
Capcase3
Cleaved-Caspase3
ns
ns
ns
**
*
**
ns
ns
ns
****
*
****
