# Supplementary material for: LRP8-dependent cholesterol metabolism modulates mTORC1 signaling and apoptotic pathways in multiple myeloma
Source: Cell Death Dis. 2025 Apr 8;16(1):263. doi: 10.1038/s41419-025-07625-w (PMC11978852; doi:10.1038/s41419-025-07625-w)
Supplement: Supplementary file 8 — Original Data File [file 41419_2025_7625_MOESM8_ESM.pdf]

AMO1

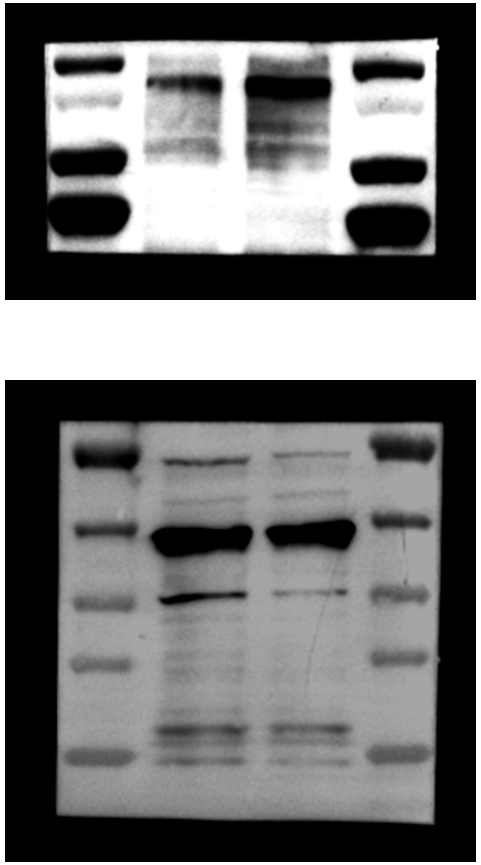

H929

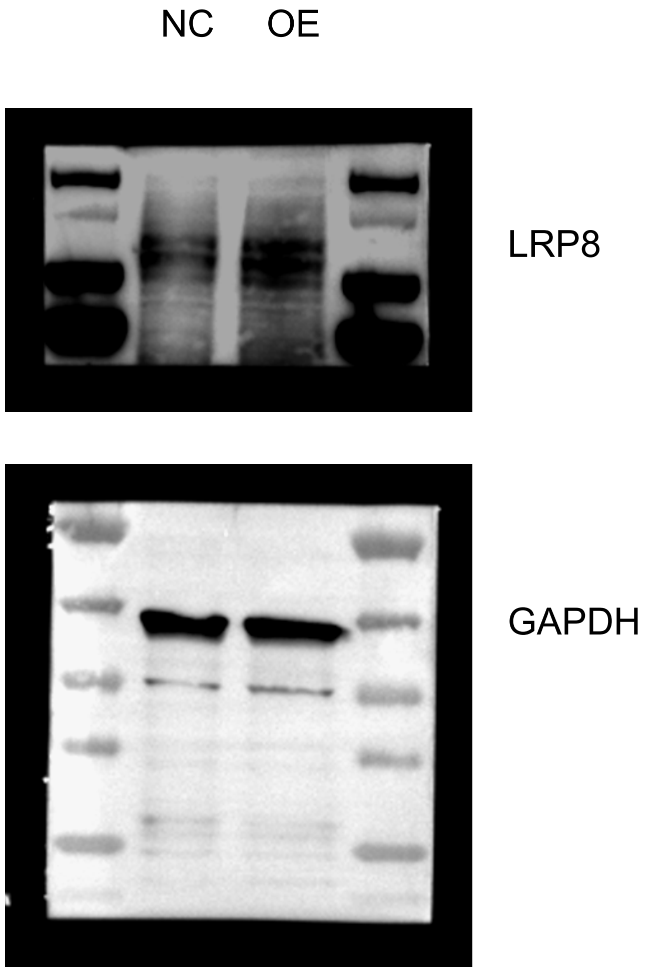

AMO1

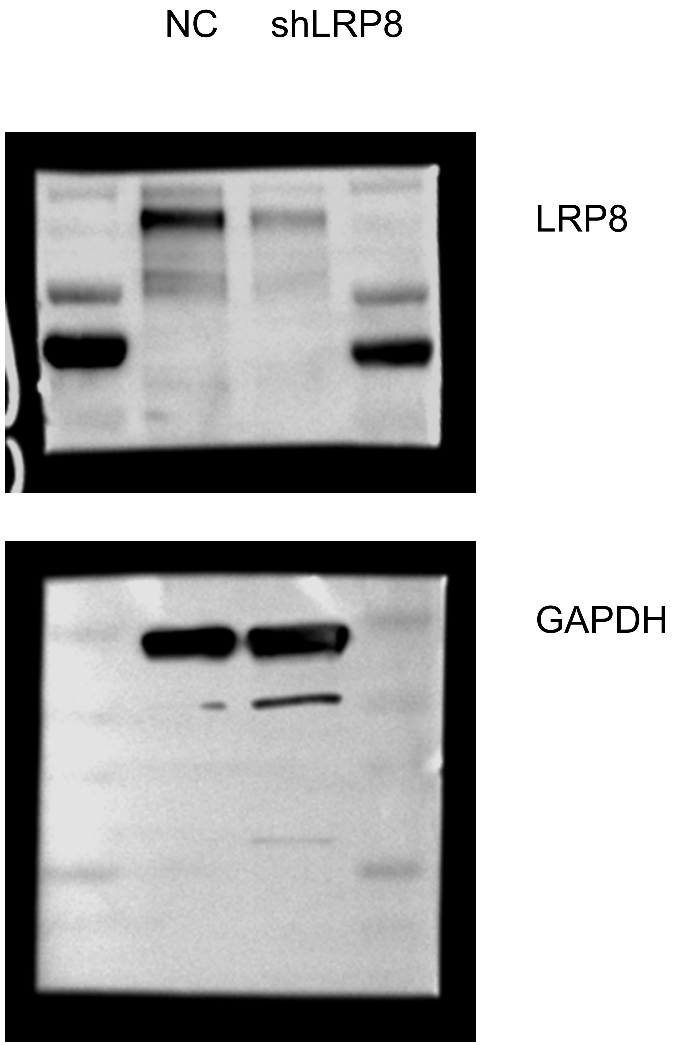

H929

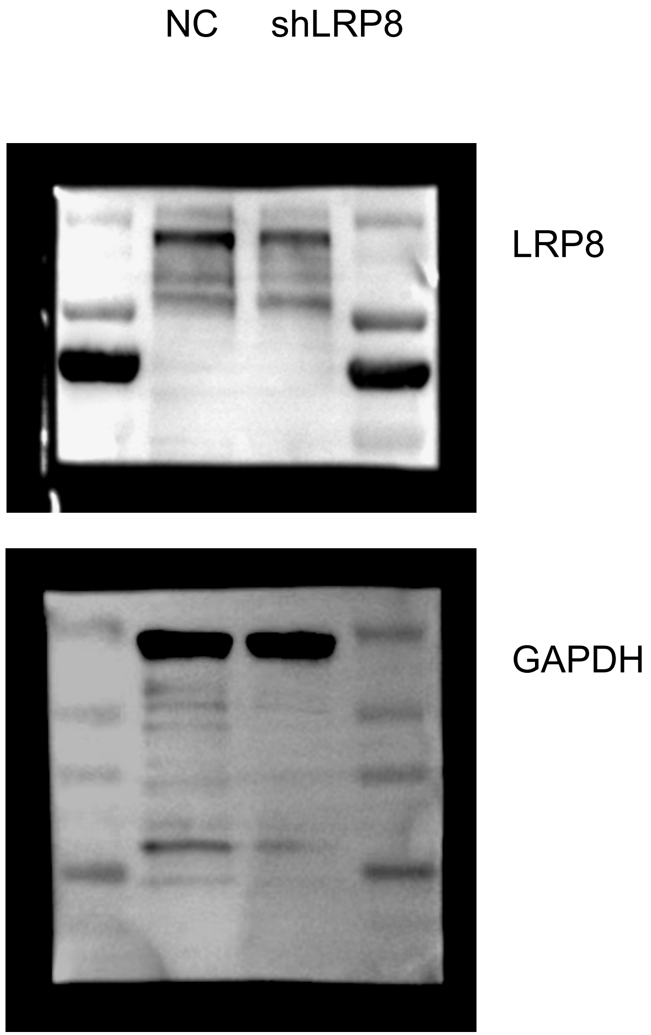

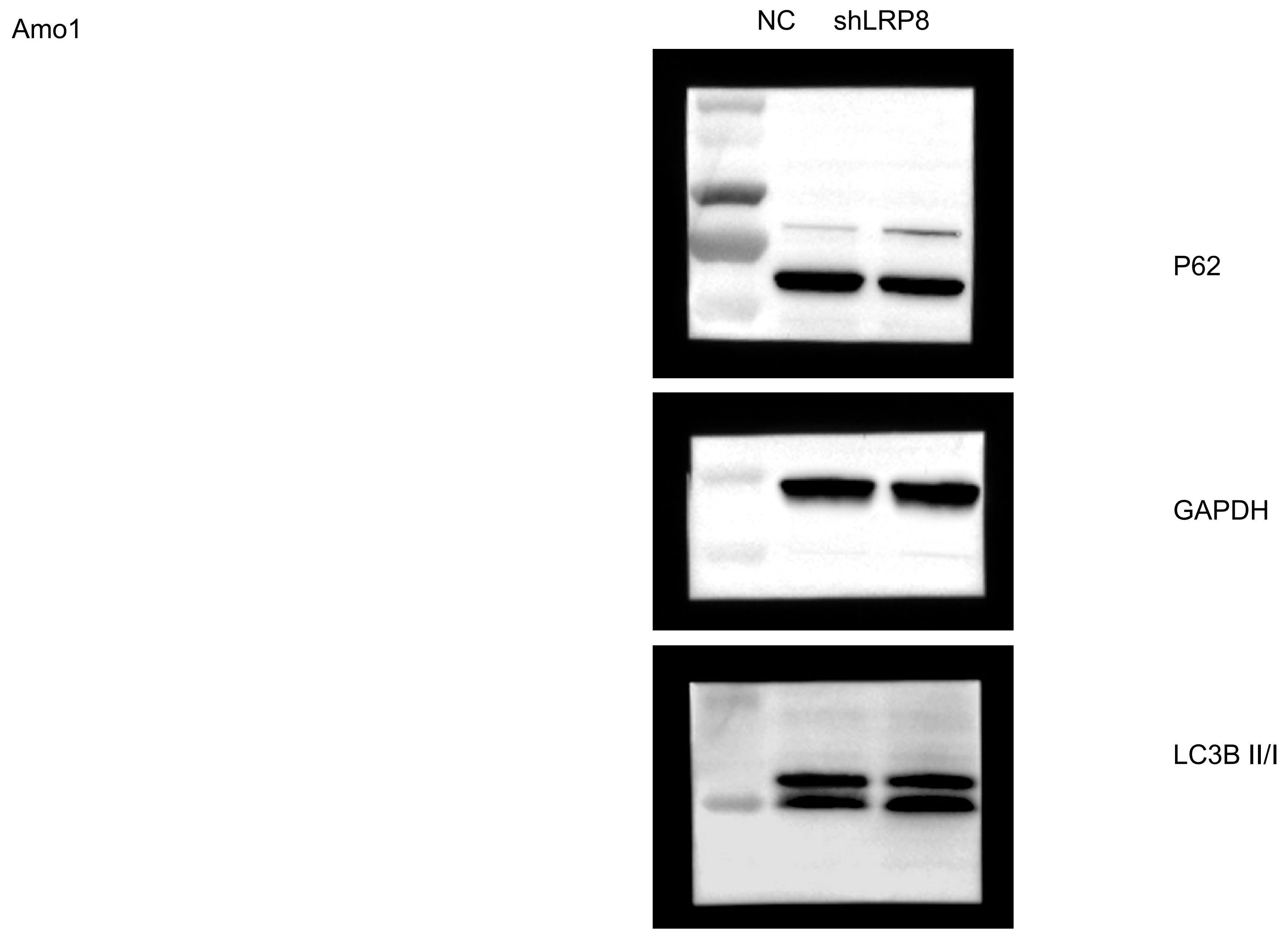

Amo1
NC
shLRP8
P62
GAPDH
LC3B II/I

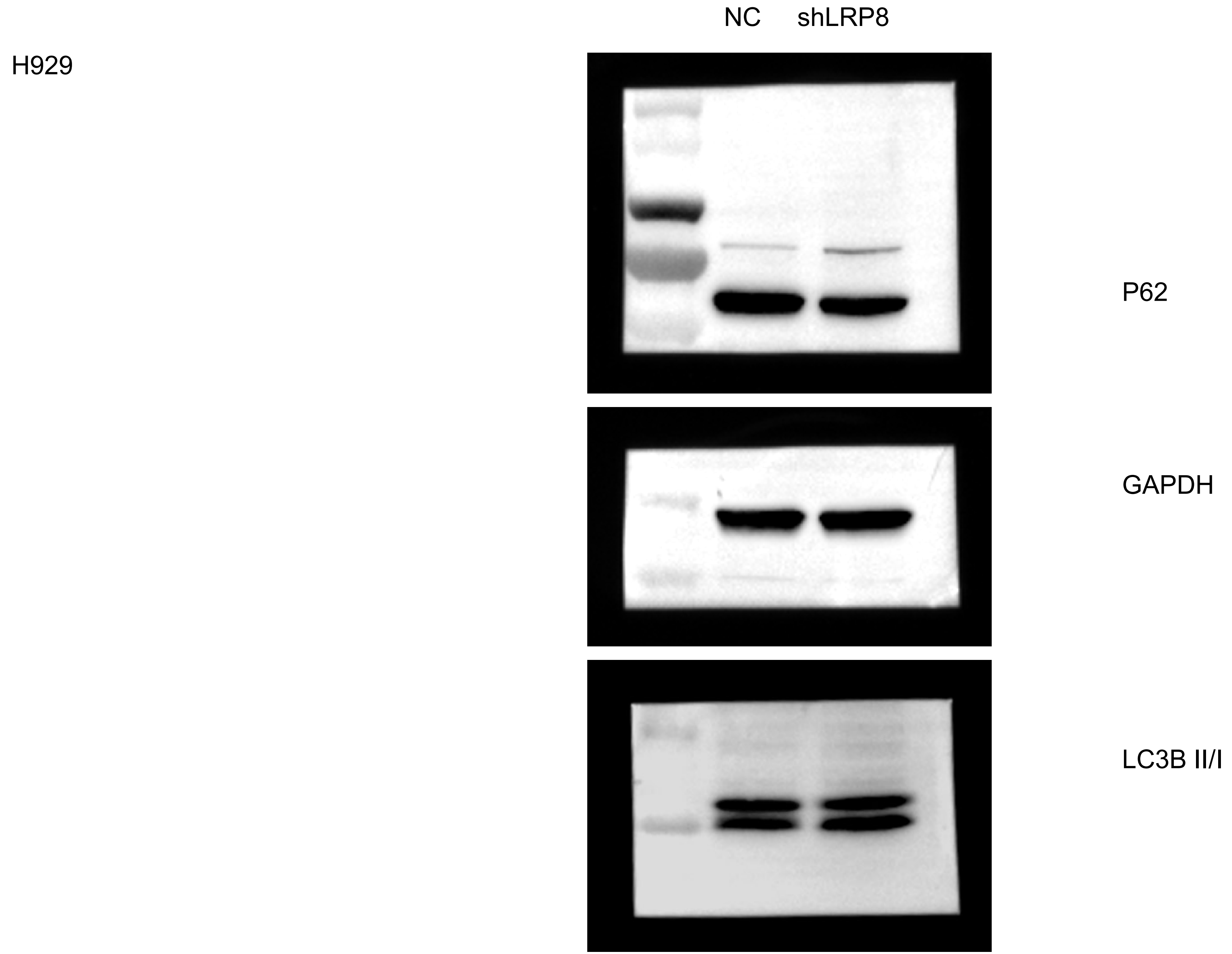
H929
NC
shLRP8
P62
GAPDH
LC3B II/I

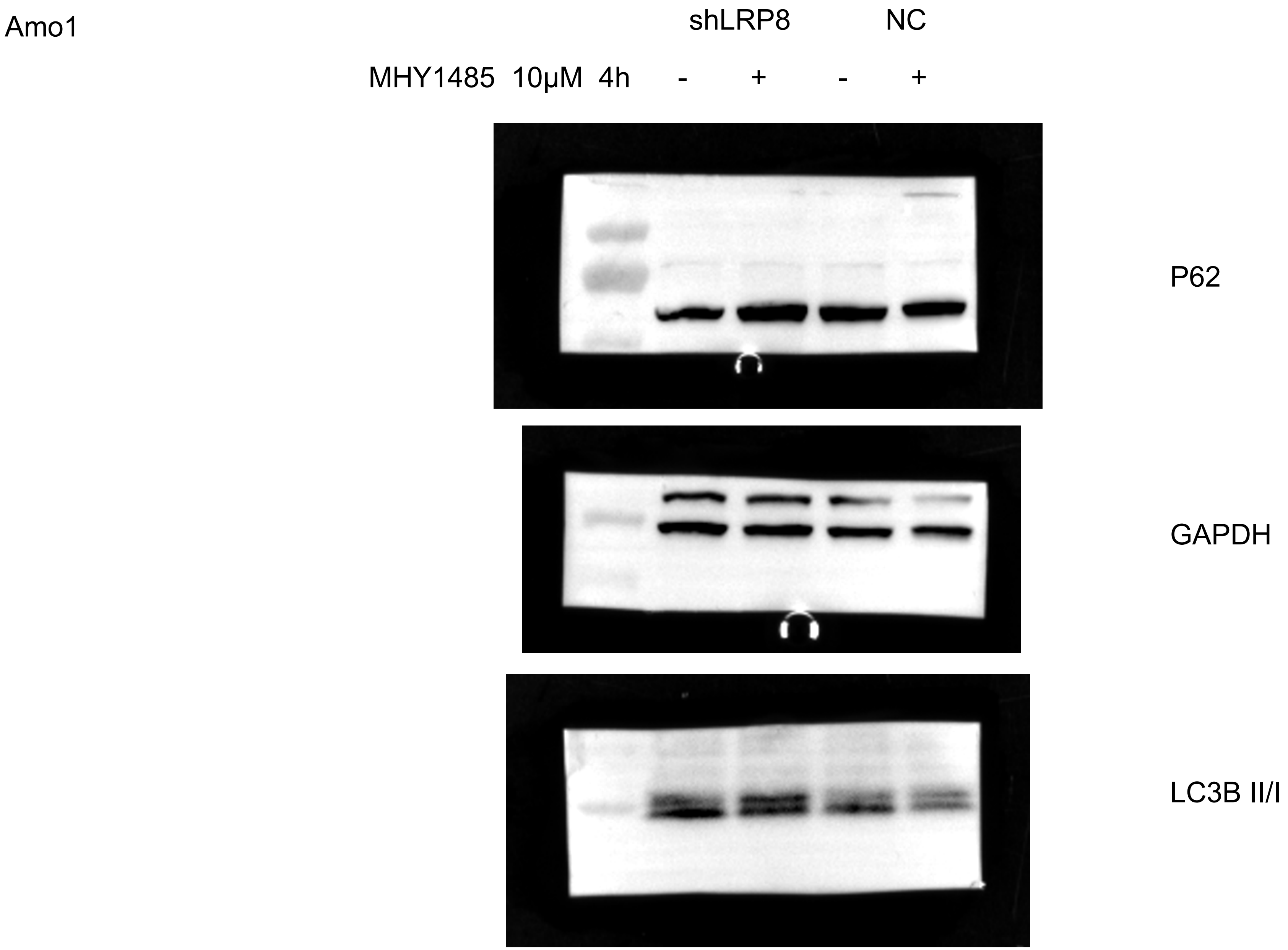

Amo1
shLRP8
NC
MHY1485 10μM 4h
-
+
-
+
P62
GAPDH
LC3B II/I

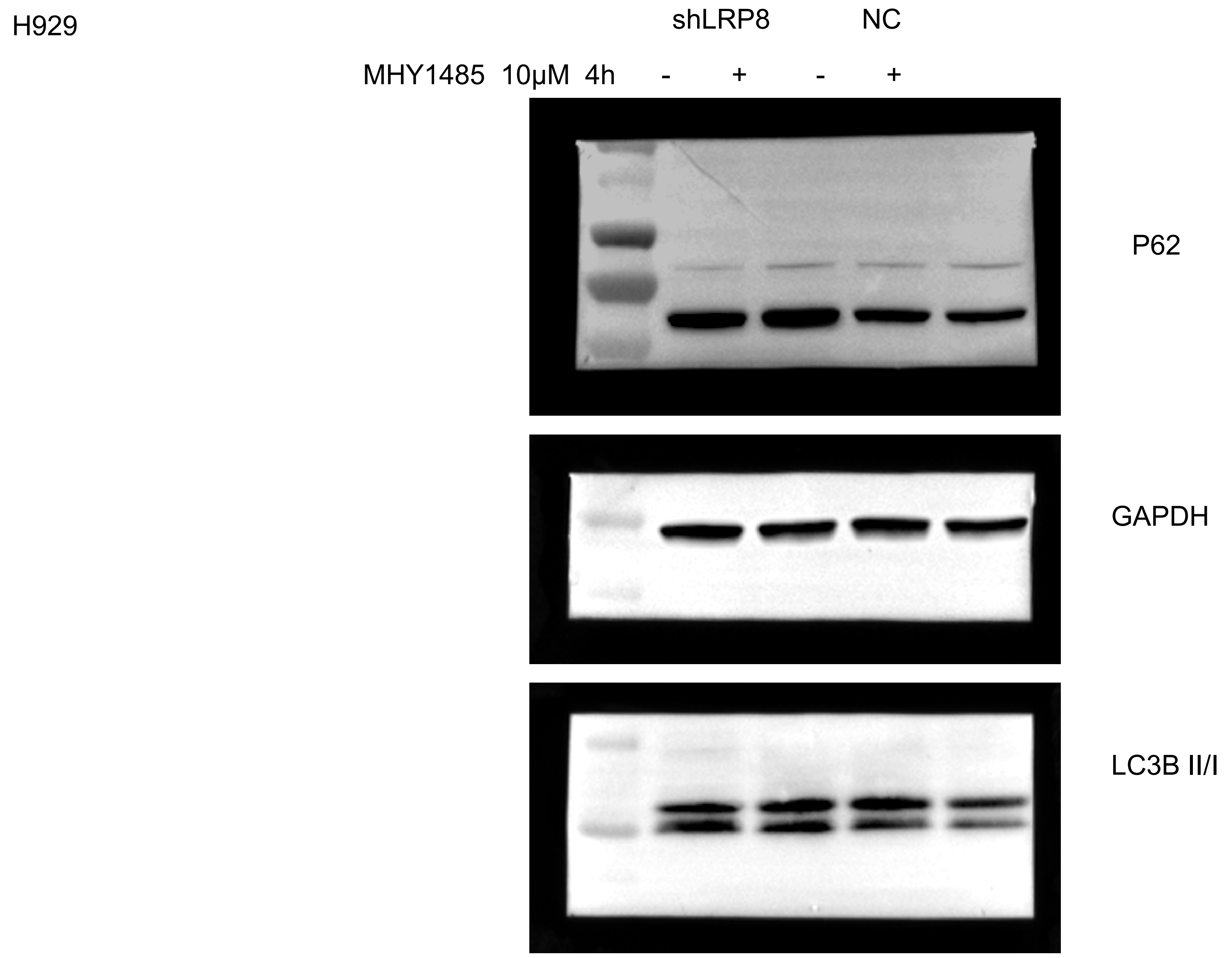
H929
shLRP8
NC
MHY1485 10μM 4h
-
+
-
+
P62
GAPDH
LC3B II/I

Amo1

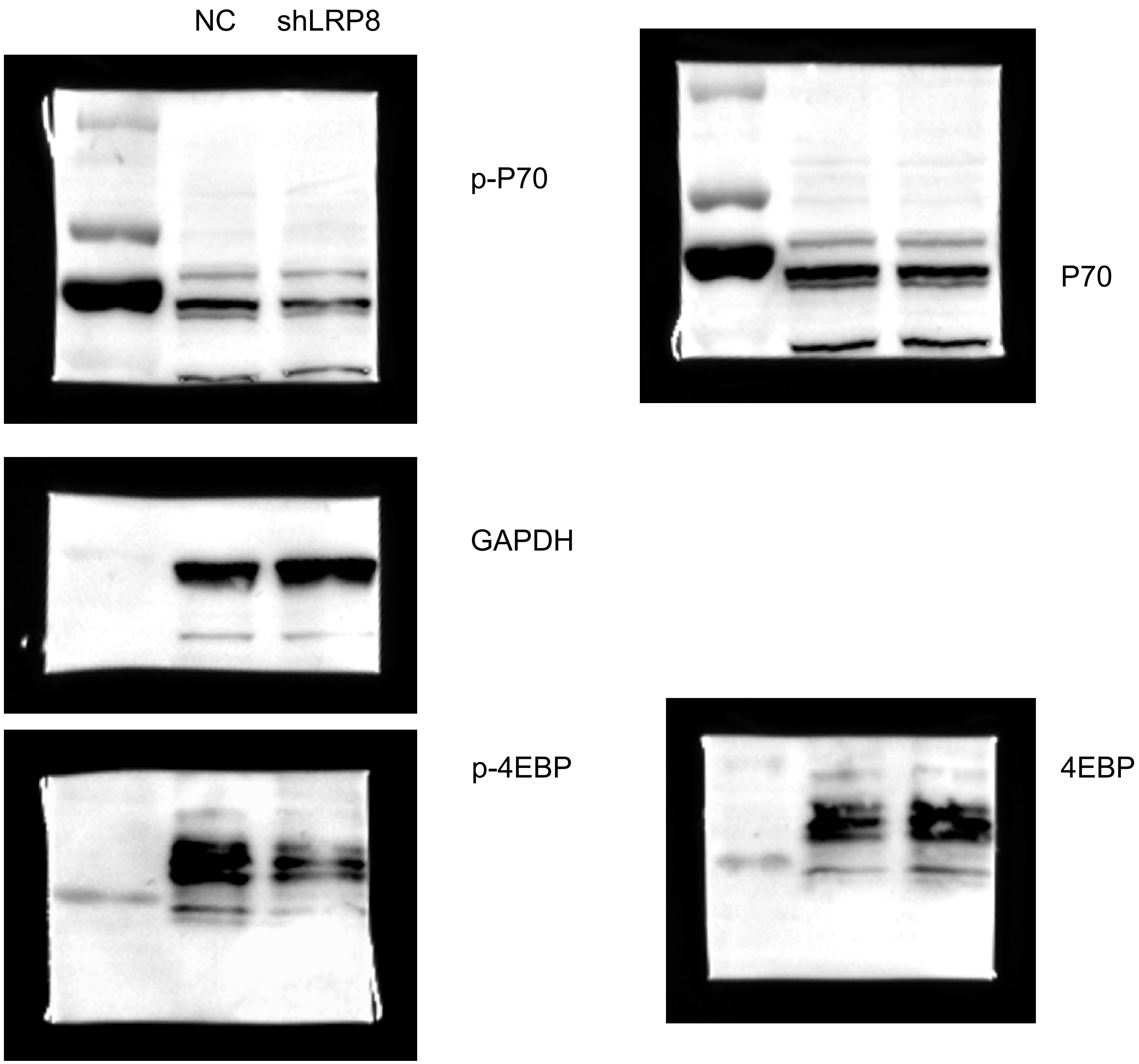

Amo1

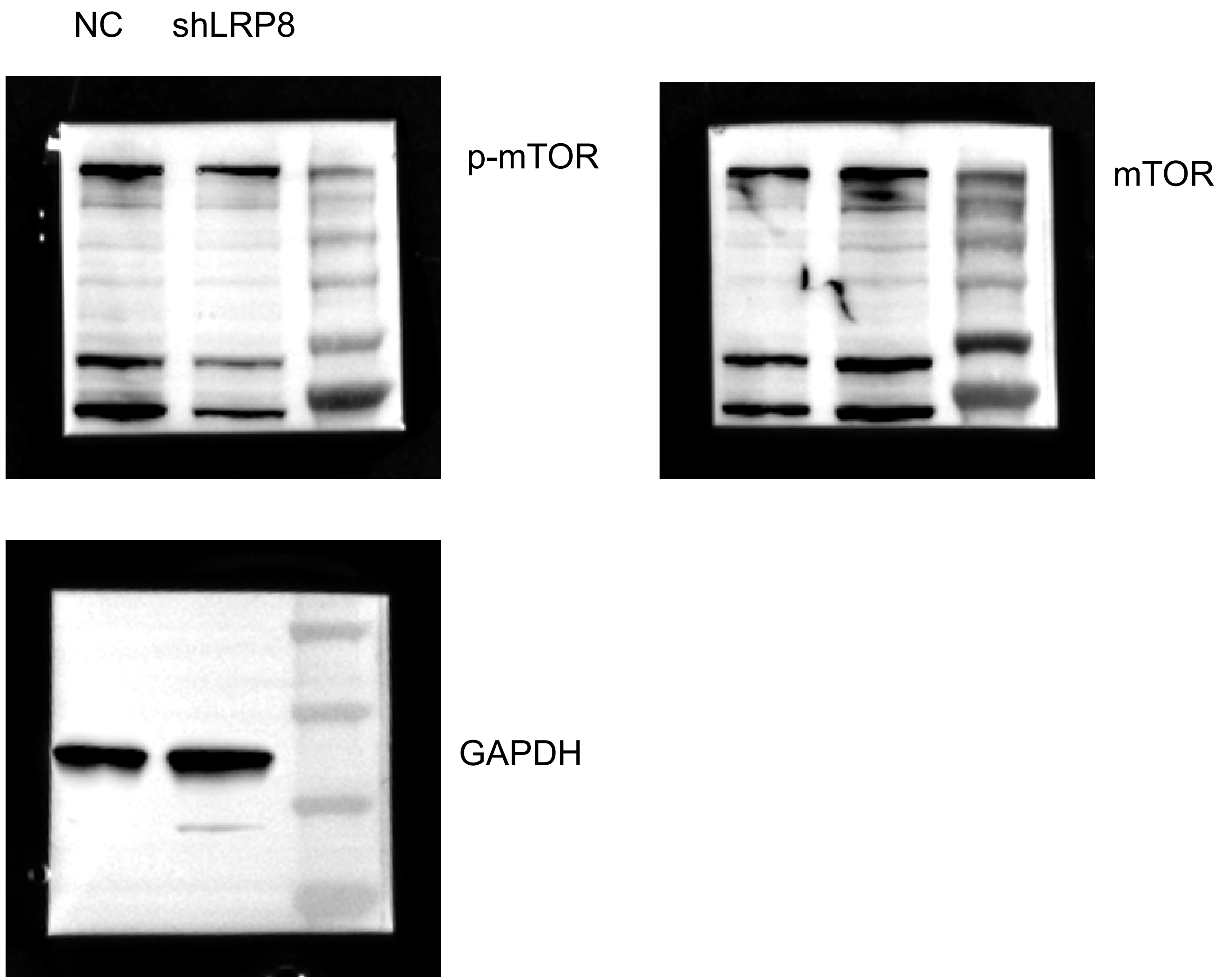

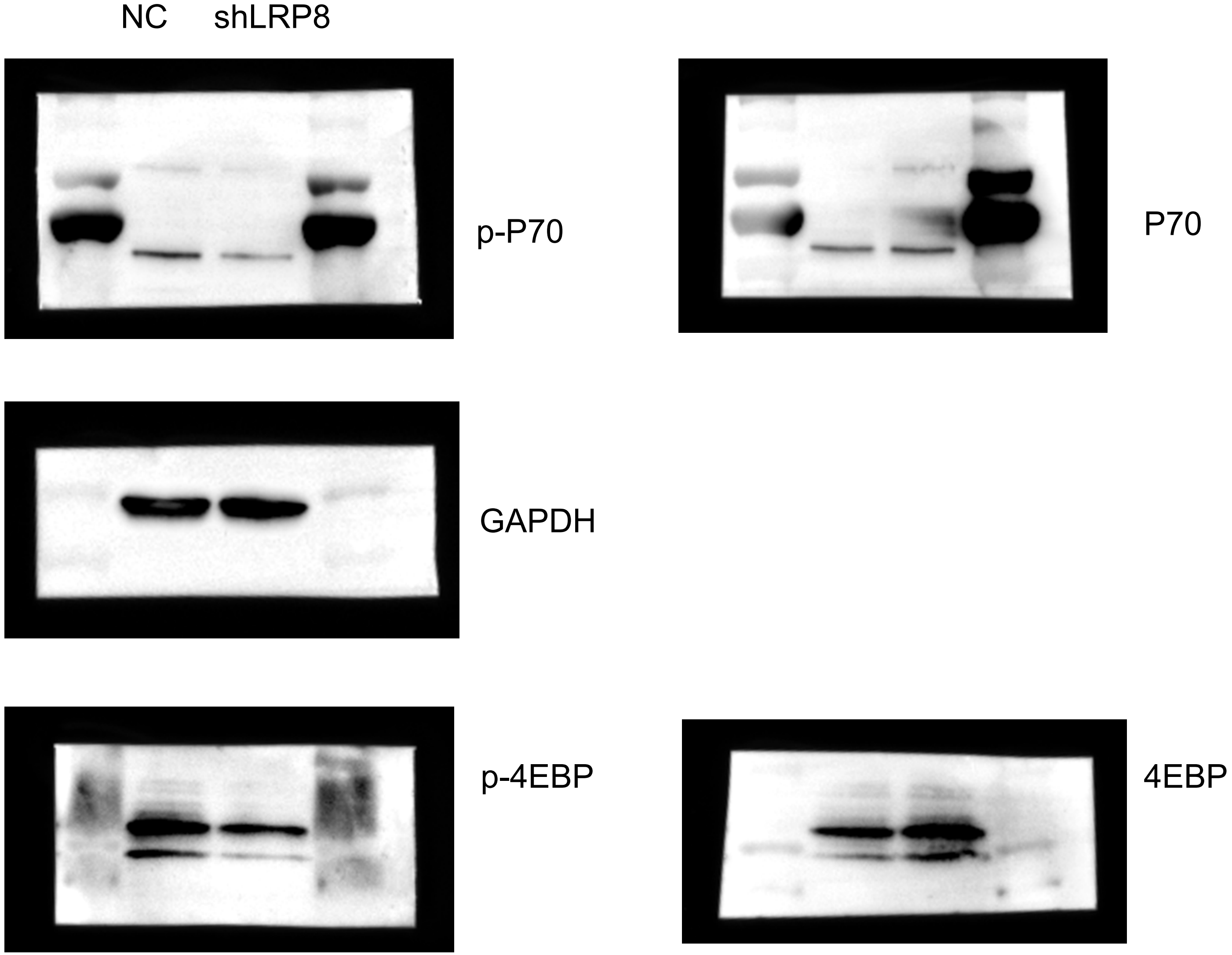
H929
NC
shLRP8
p-P70
P70
GAPDH
p-4EBP
4EBP

H929

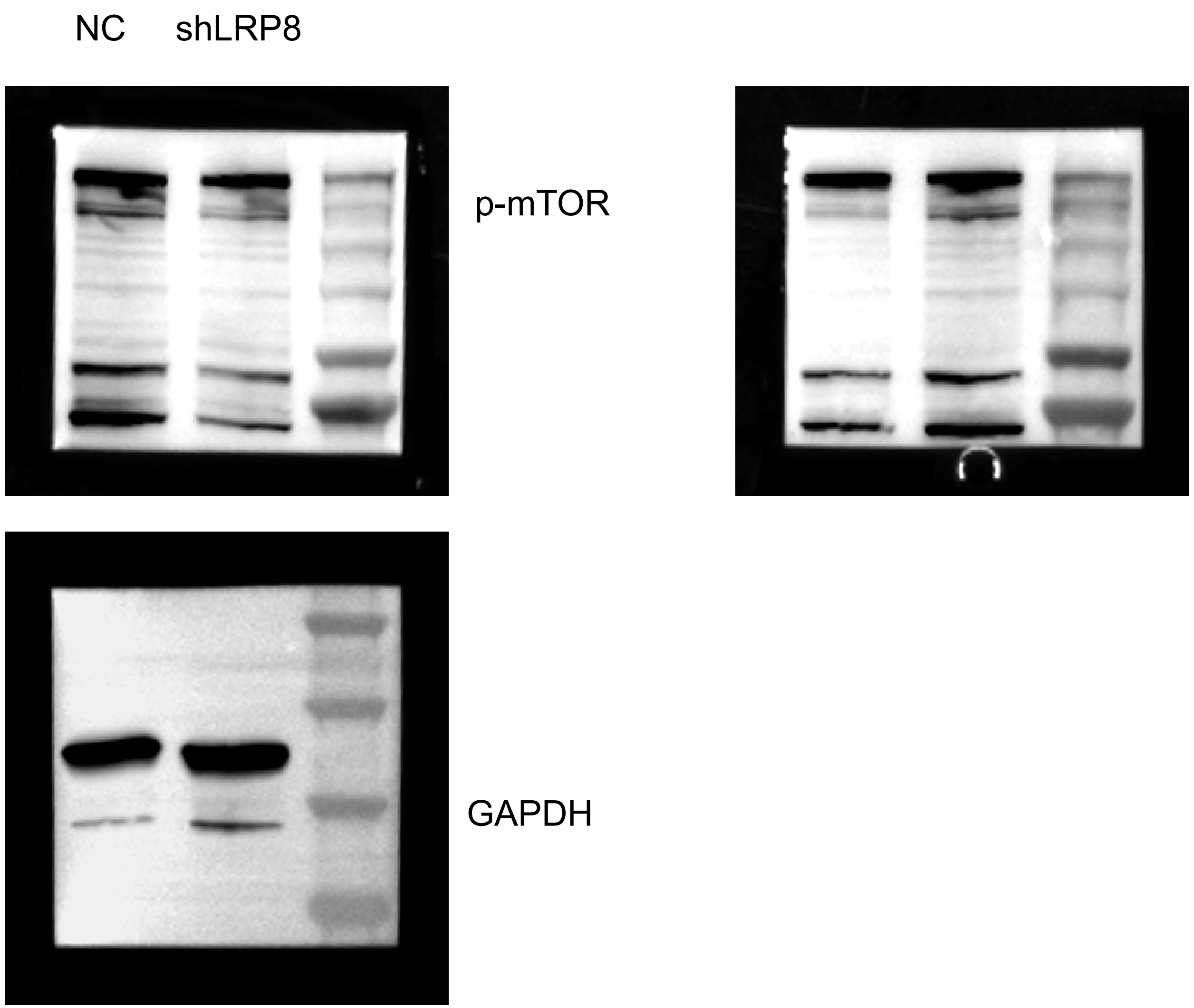

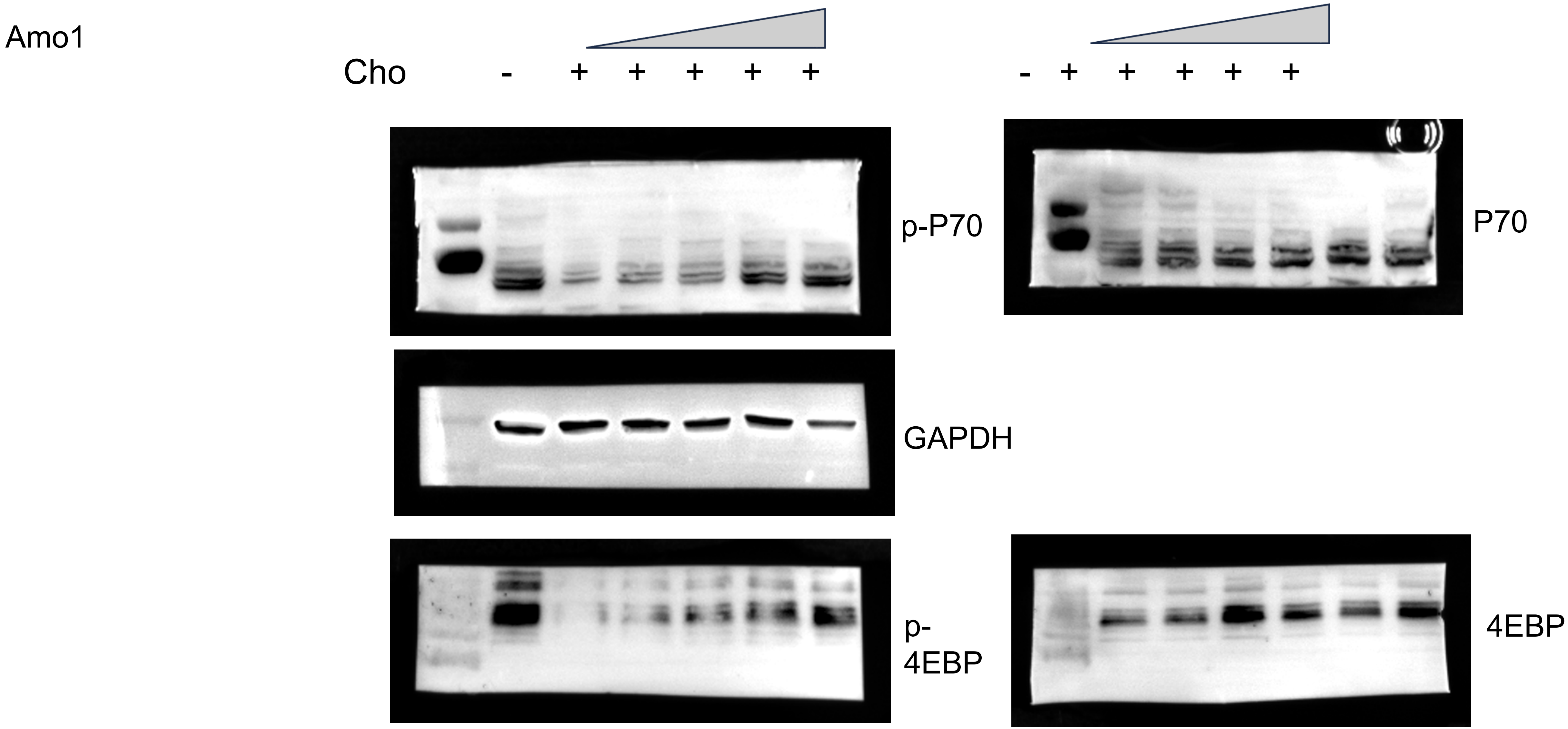

Amo1
Cho
- + + + + +
- + + + + +
p-P70
P70
GAPDH
p-
4EBP
4EBP

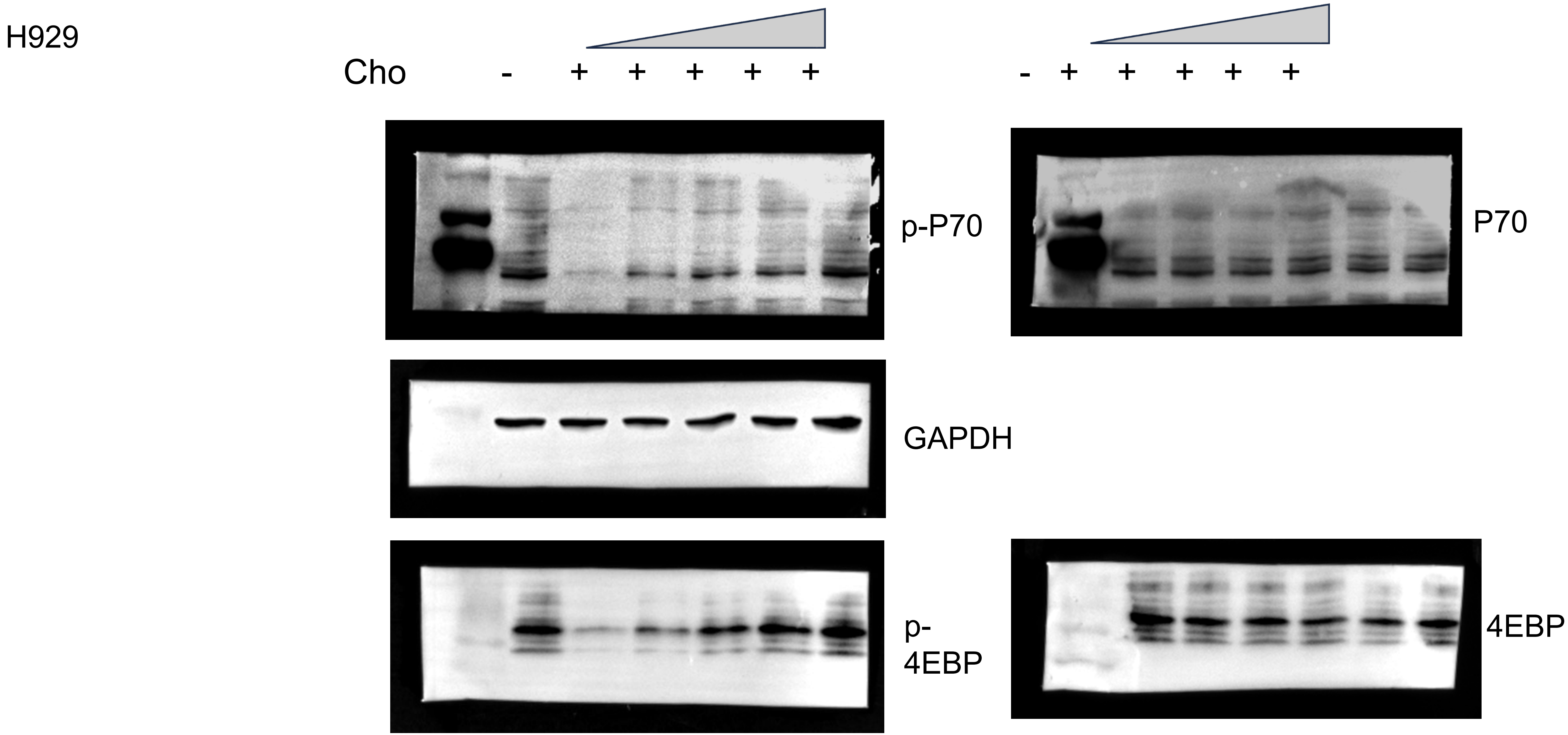
H929
Cho
- + + + + +
- + + + + +
p-P70
P70
GAPDH
p-
4EBP
4EBP

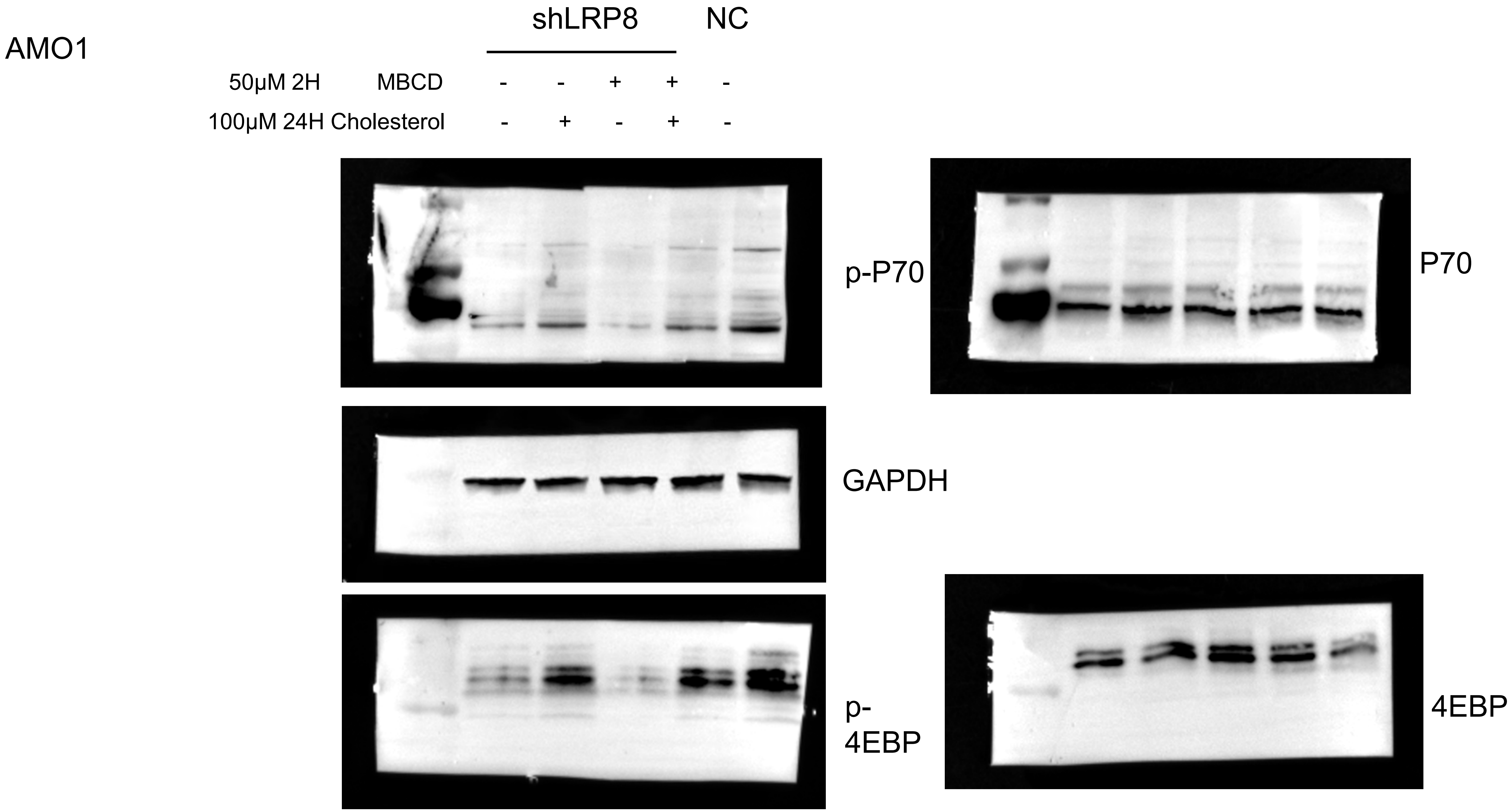
AMO1
shLRP8
NC
50µM 2H MBCD - - + + -
100µM 24H Cholesterol - + - + -
p-P70
P70
GAPDH
p-4EBP
4EBP

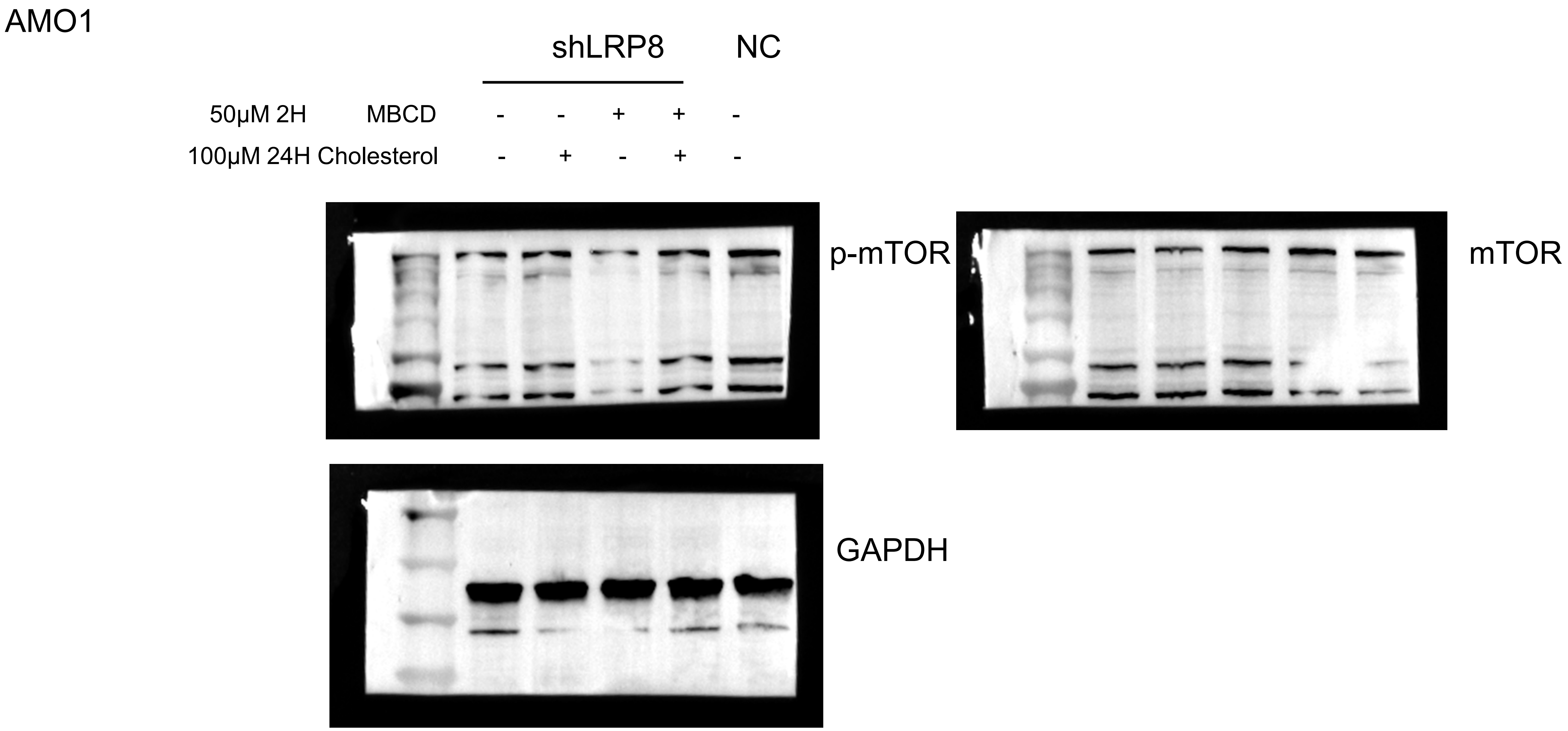
AMO1
shLRP8
NC
50μM 2H MBCD - - + + -
100μM 24H Cholesterol - + - + -
p-mTOR
mTOR
GAPDH

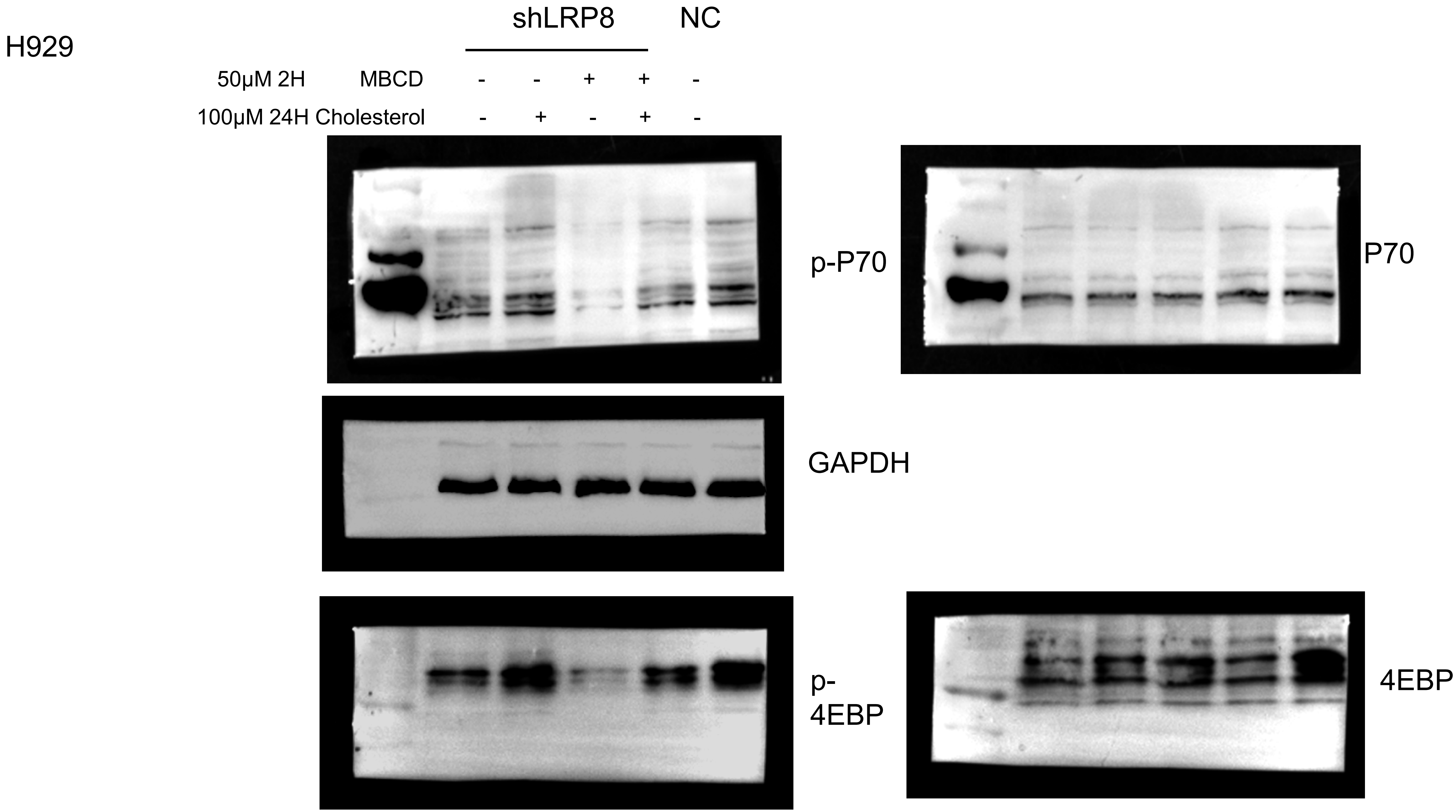
H929
shLRP8
NC
50μM 2H MBCD
- - + + -
100μM 24H Cholesterol
- + - + -
p-P70
P70
GAPDH
p-4EBP
4EBP

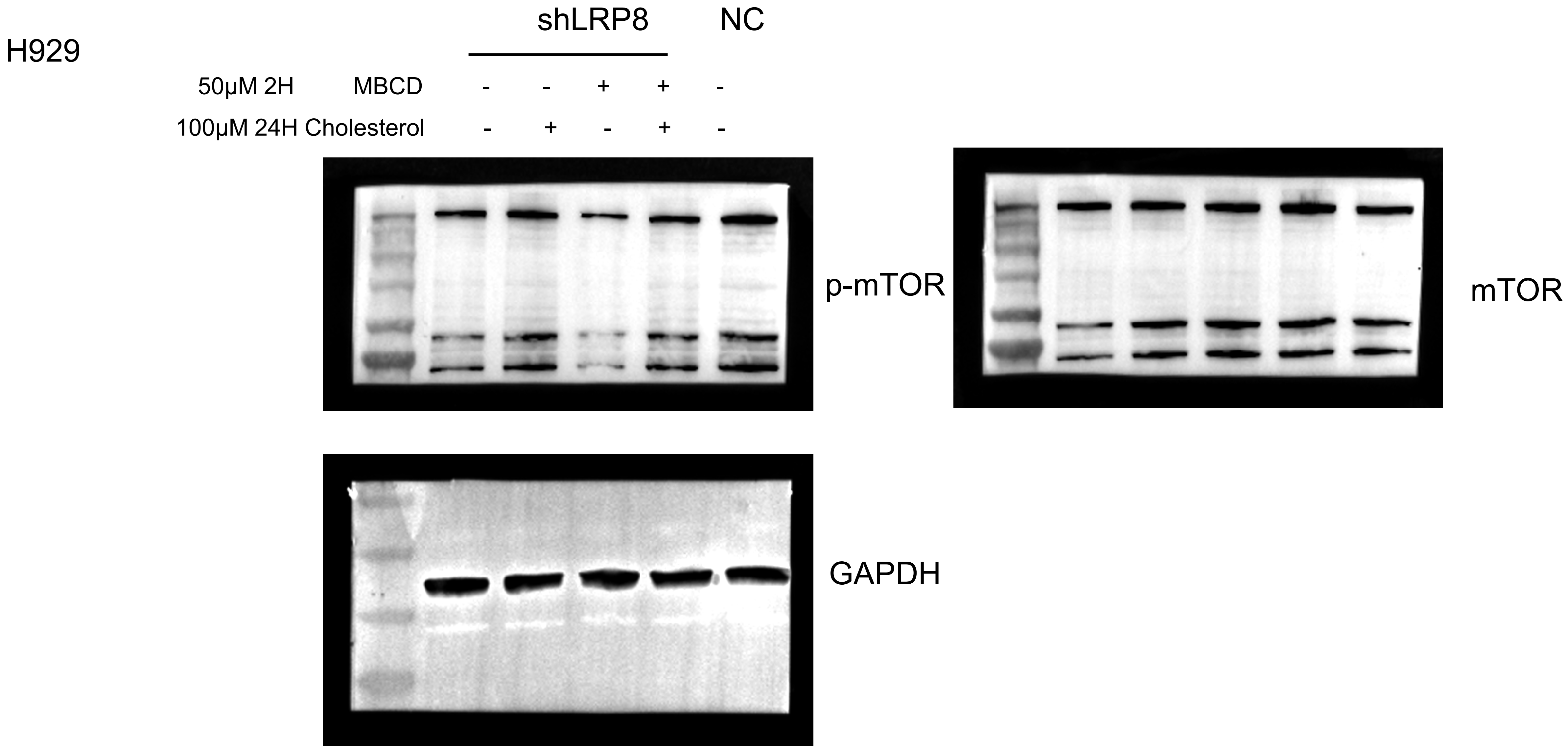

H929
shLRP8
NC
50μM 2H MBCD - - + + -
100μM 24H Cholesterol - + - + -
p-mTOR
mTOR
GAPDH

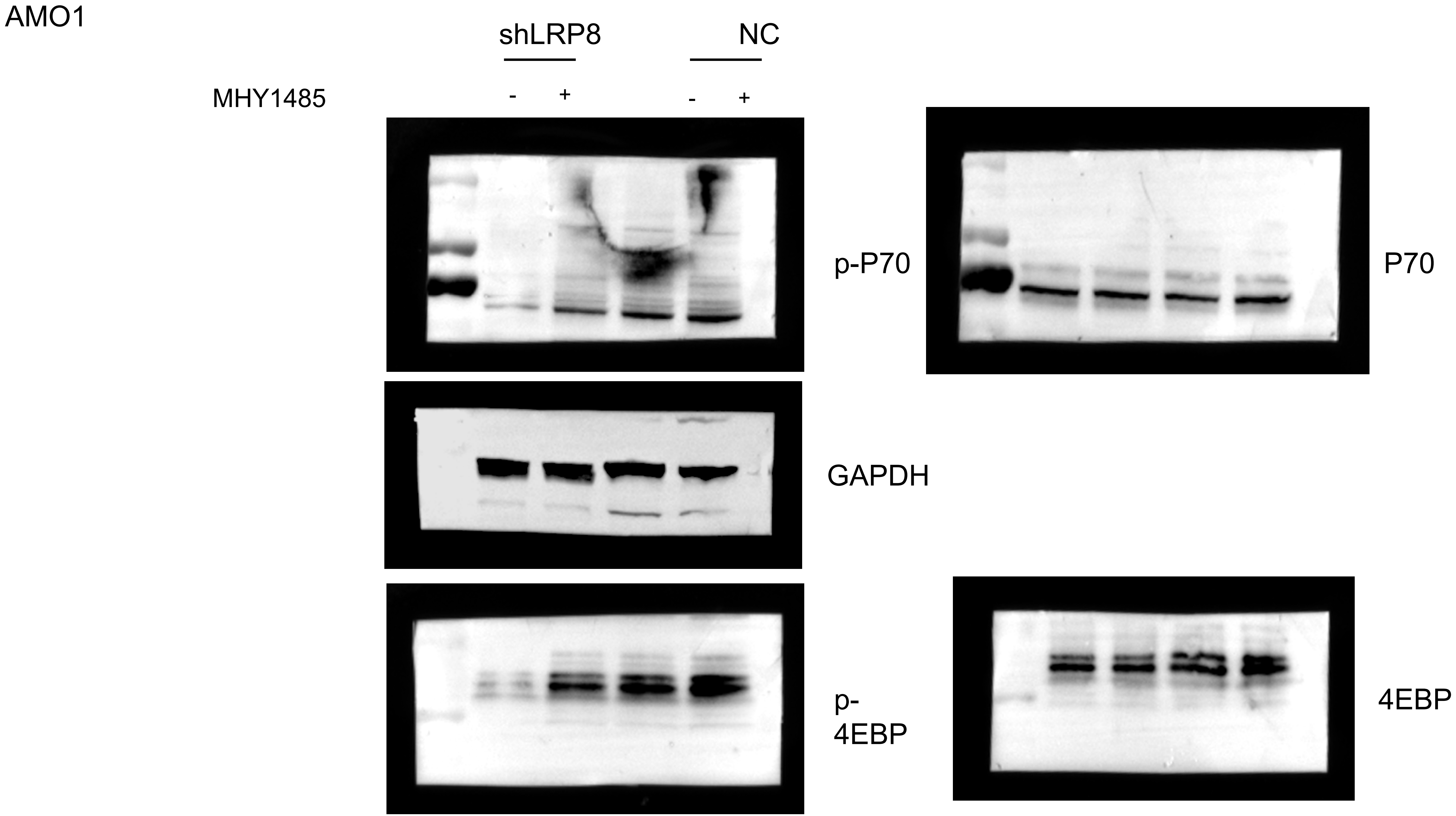
AMO1
shLRP8
NC
MHY1485
-
+
-
+
p-P70
P70
GAPDH
p-
4EBP
4EBP

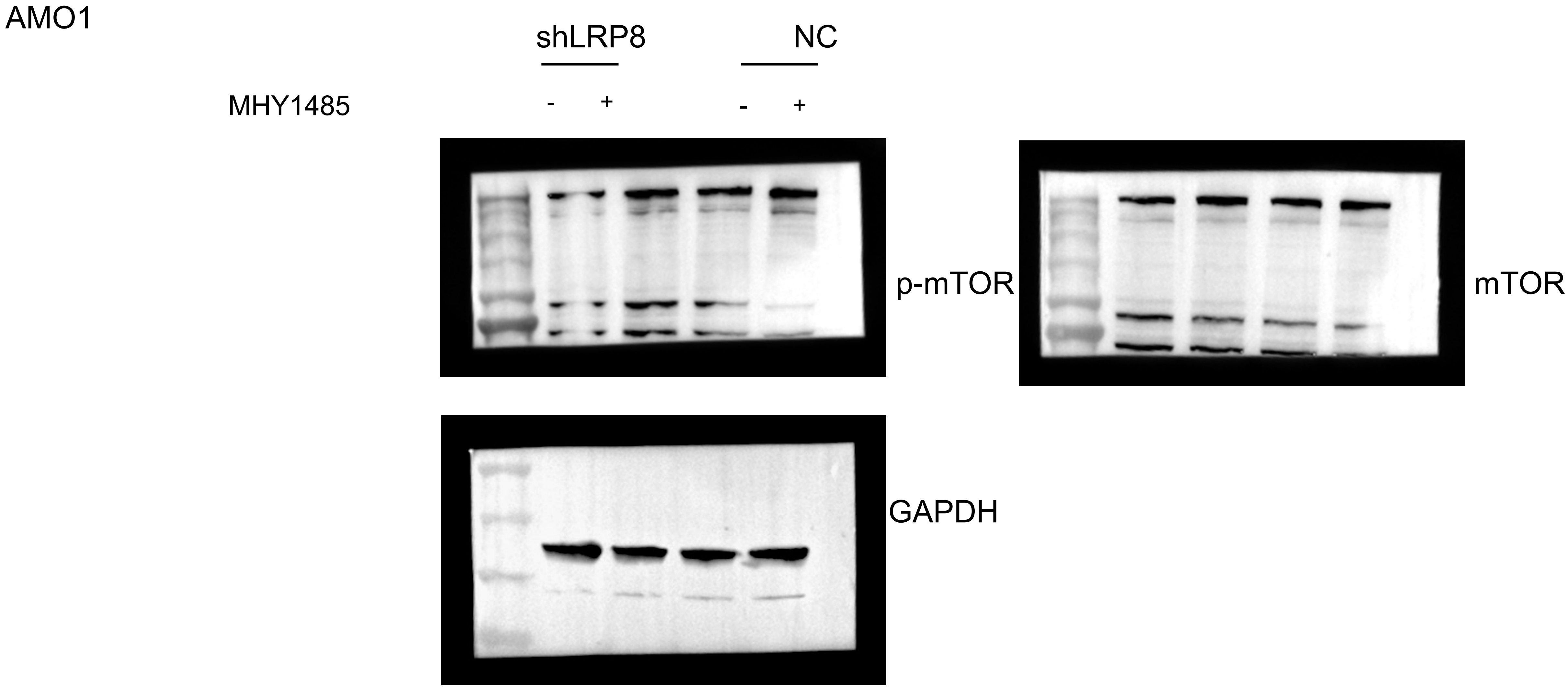
AMO1
shLRP8
NC
MHY1485
-
+
-
+
p-mTOR
mTOR
GAPDH

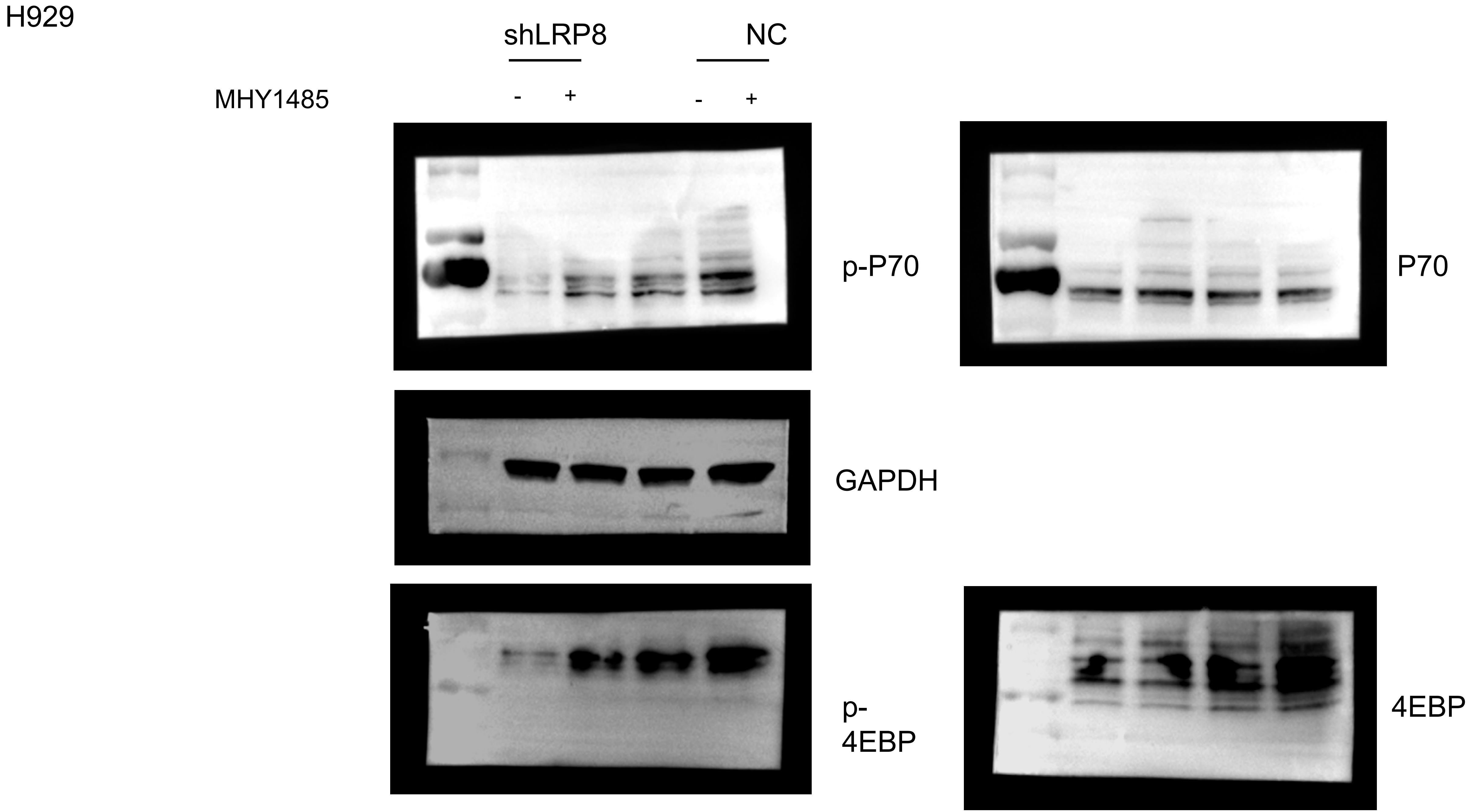
H929
shLRP8
NC
MHY1485
-
+
-
+
p-P70
P70
GAPDH
p-
4EBP
4EBP

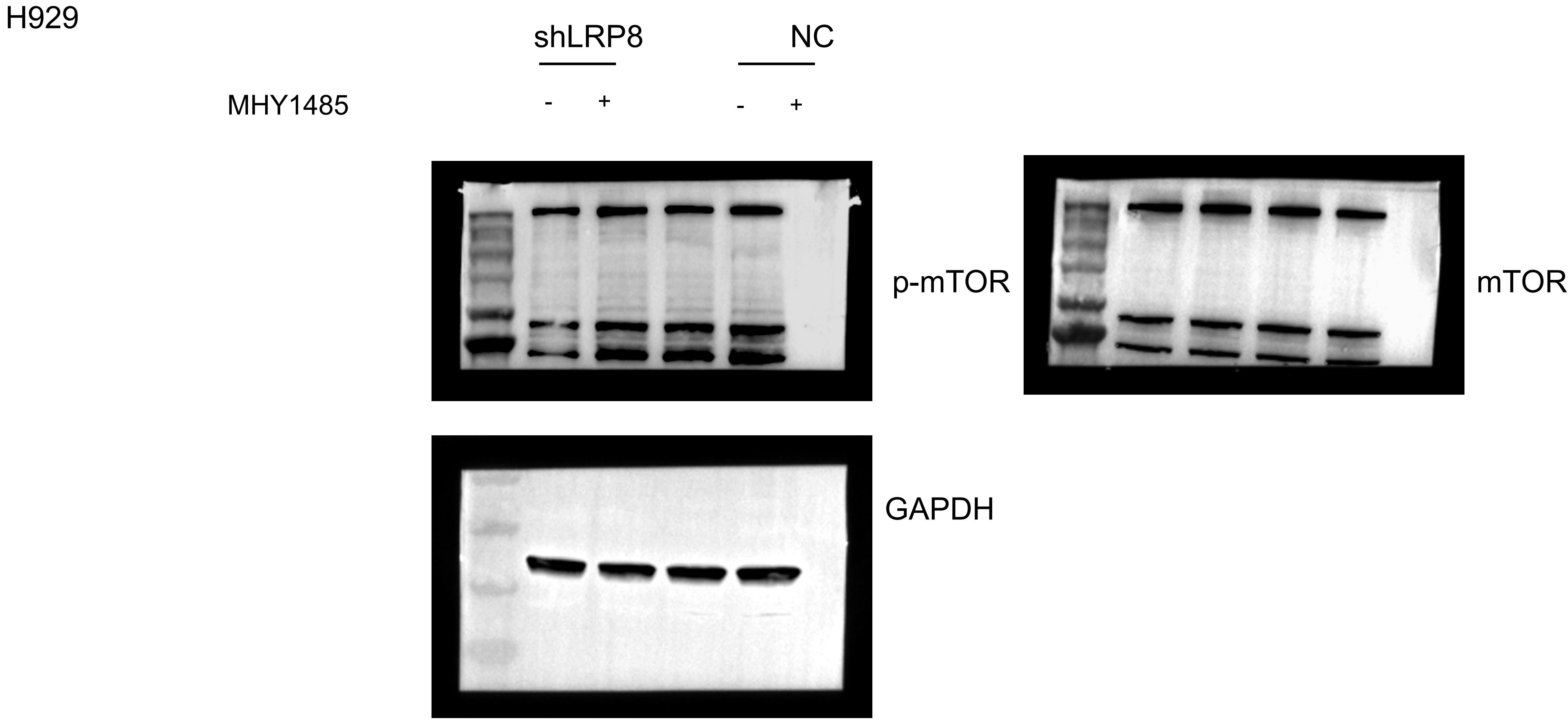
H929
shLRP8
NC
MHY1485
-
+
-
+
p-mTOR
mTOR
GAPDH

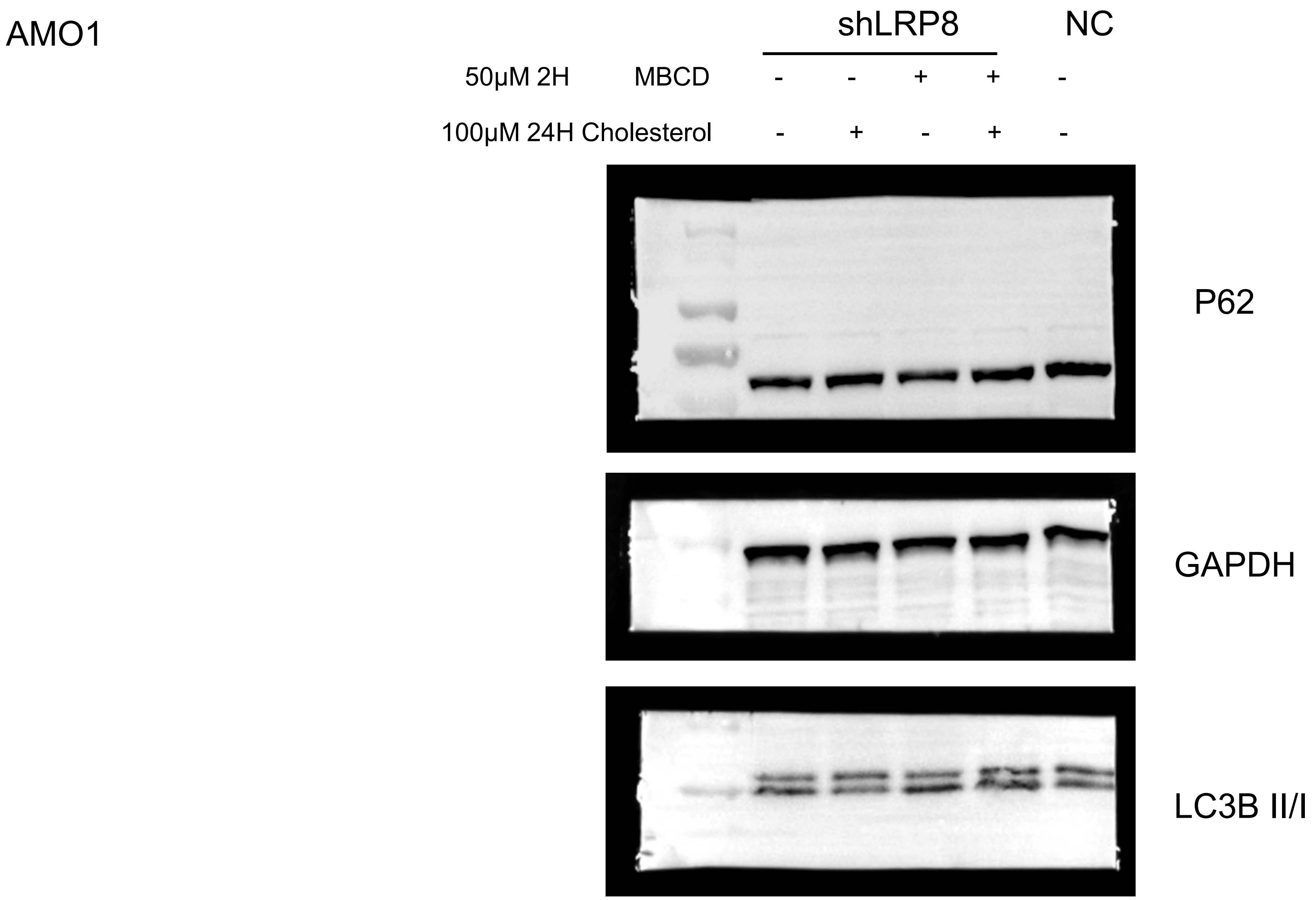
AMO1
shLRP8
NC
50μM 2H MBCD - - + + -
100μM 24H Cholesterol - + - + -
P62
GAPDH
LC3B II/I

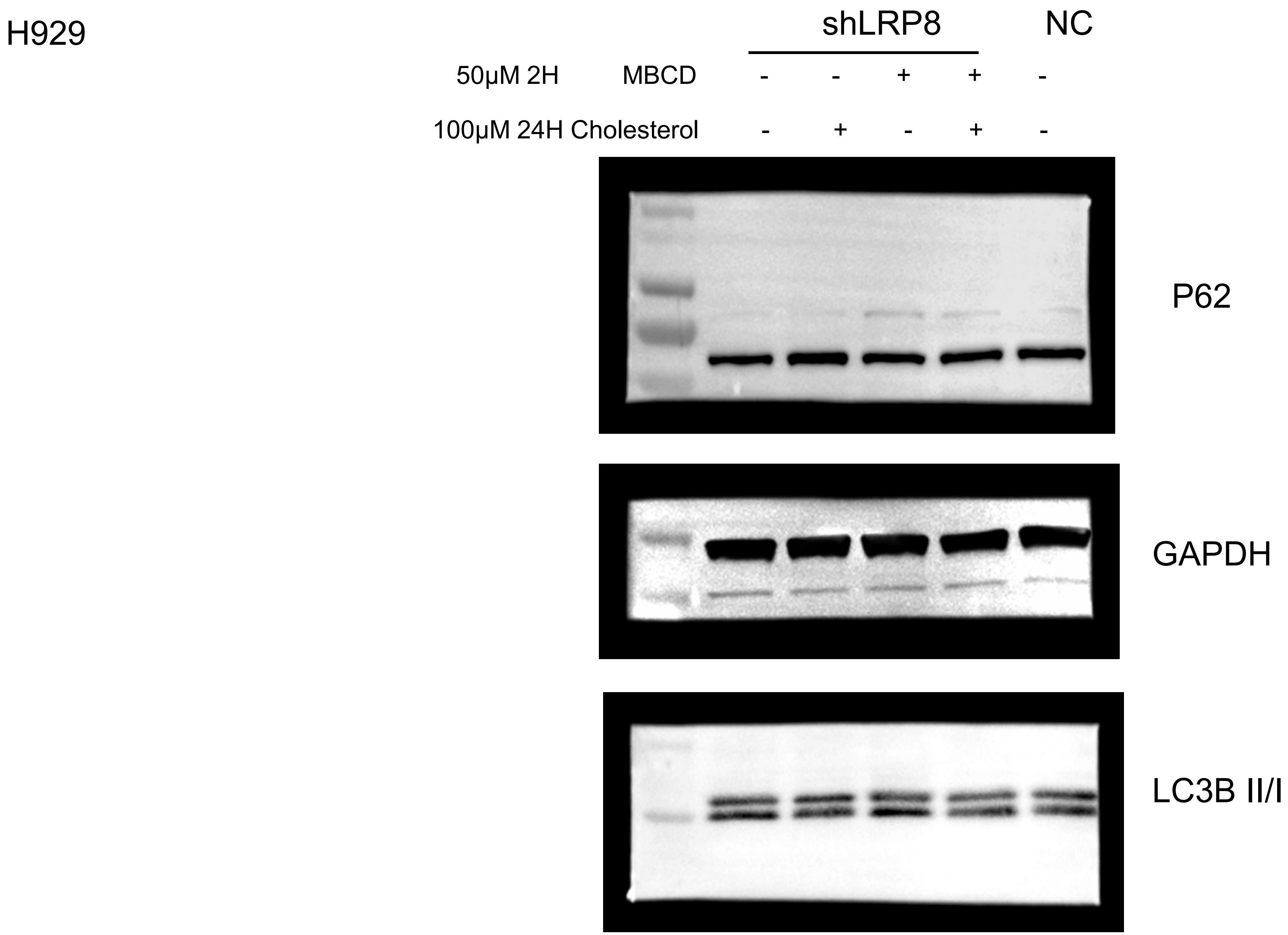
H929
shLRP8
NC
50μM 2H MBCD - - + + -
100μM 24H Cholesterol - + - + -
P62
GAPDH
LC3B II/I

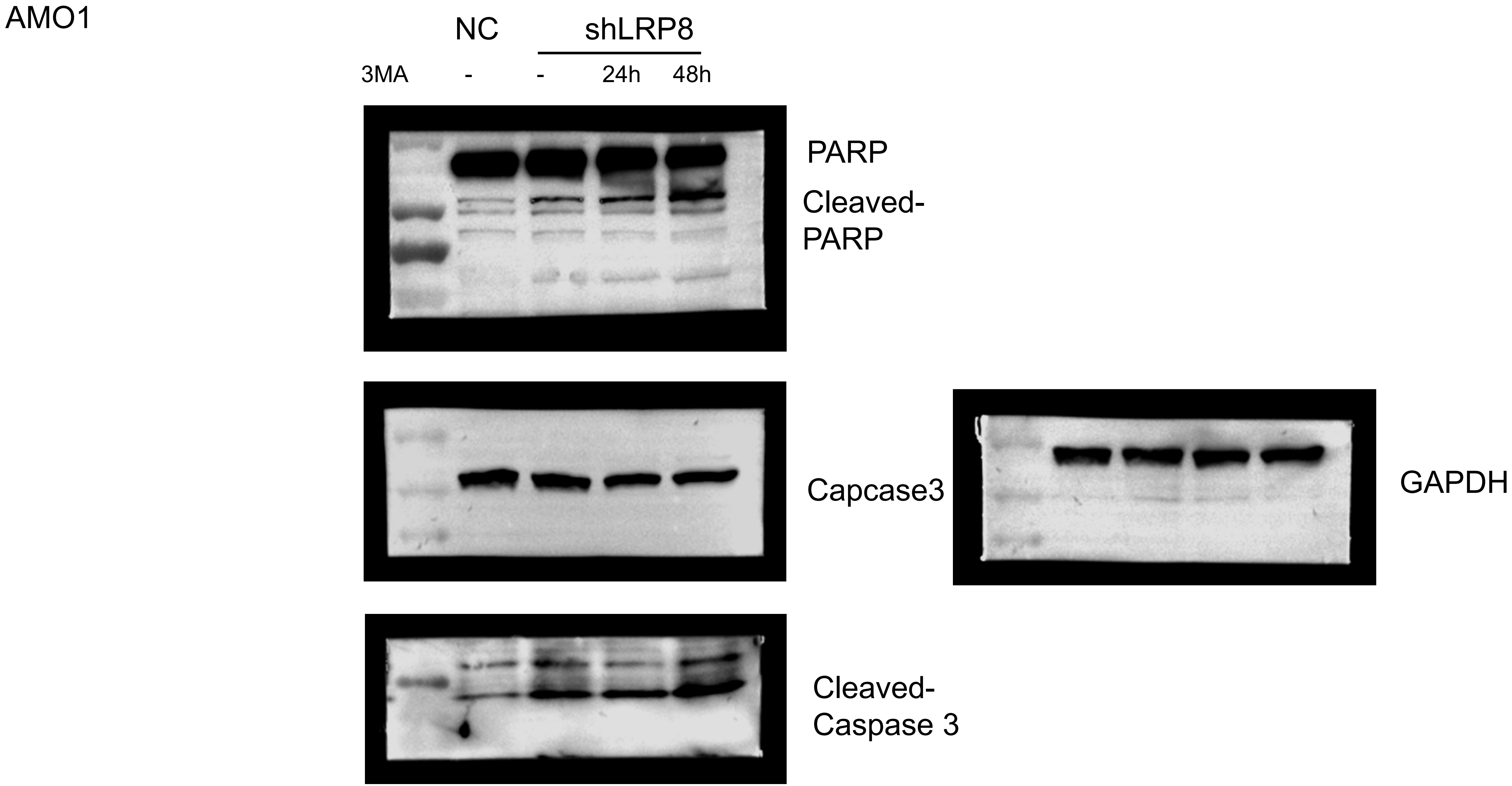
AMO1
NC
shLRP8
3MA
-
-
24h
48h
PARP
Cleaved-
PARP
Capcase3
GAPDH
Cleaved-
Caspase 3

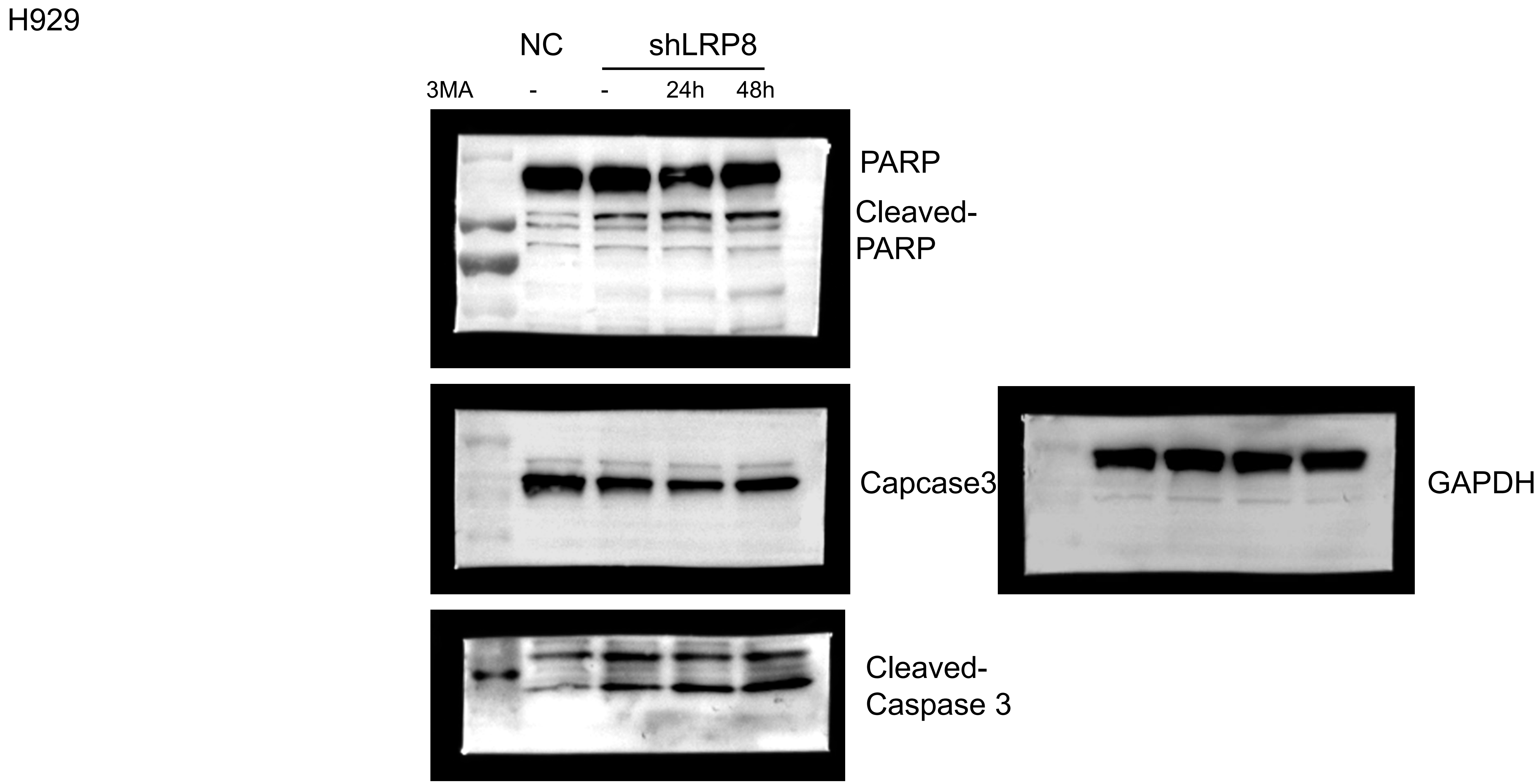
H929
NC
shLRP8
3MA
-
-
24h
48h
PARP
Cleaved-
PARP
Capcase3
GAPDH
Cleaved-
Caspase 3
